# Enhancing calmodulin binding to ryanodine receptor is crucial to limit neuronal cell loss in Alzheimer disease

**DOI:** 10.1038/s41598-021-86822-x

**Published:** 2021-03-31

**Authors:** Yoshihide Nakamura, Takeshi Yamamoto, Xiaojuan Xu, Shigeki Kobayashi, Shinji Tanaka, Masaki Tamitani, Takashi Saito, Takaomi C. Saido, Masafumi Yano

**Affiliations:** 1 Department of Medicine and Clinical Science, Division of Cardiology, Yamaguchi University Graduate School of Medicine, 1-1-1 Minamikogushi, Ube, Yamaguchi 755-8505 Japan; 2 Faculty of Health Sciences, Yamaguchi University Graduate School of Medicine, Ube, Japan; 3 Department of Pathology and Pathophysiology, School of Medicine, Tongji University, Shanghai, China; 4 Department of Neurocognitive Science, Institute of Brain Science, Nagoya City University Graduate School of Medical Science, Nagoya, Japan; 5 RIKEN Center for Brain Science, Laboratory for Proteolytic Neuroscience, Wako, Japan

**Keywords:** Biophysics, Cell biology, Neuroscience

## Abstract

Alzheimer’s disease (AD) is a neurodegenerative disorder characterized by progressive neuronal cell loss. Recently, dysregulation of intracellular Ca^2+^ homeostasis has been suggested as a common proximal cause of neural dysfunction in AD. Here, we investigated (1) the pathogenic role of destabilization of ryanodine receptor (RyR2) in endoplasmic reticulum (ER) upon development of AD phenotypes in *App*^*NL-G-F*^ mice, which harbor three familial AD mutations (Swedish, Beyreuther/Iberian, and Arctic), and (2) the therapeutic effect of enhanced calmodulin (CaM) binding to RyR2. In the neuronal cells from *App*^*NL-G-F*^ mice, CaM dissociation from RyR2 was associated with AD-related phenotypes, i.e. Aβ accumulation, TAU phosphorylation, ER stress, neuronal cell loss, and cognitive dysfunction. Surprisingly, either genetic (by V3599K substitution in RyR2) or pharmacological (by dantrolene) enhancement of CaM binding to RyR2 reversed almost completely the aforementioned AD-related phenotypes, except for Aβ accumulation. Thus, destabilization of RyR2 due to CaM dissociation is most likely an early and fundamental pathogenic mechanism involved in the development of AD. The discovery that neuronal cell loss can be fully prevented simply by stabilizing RyR2 sheds new light on the treatment of AD.

## Introduction

Alzheimer’s disease (AD) is the most common type of senile dementia, characterized by alterations in memory formation and storage. Regarding the pathogenic mechanism, the amyloid hypothesis has been widely recognized, according to which, amyloid beta (Aβ) abnormally accumulates in the neural system due to a genetic disorder that influences the amyloid precursor protein and over-activation of β- and γ-secretase, leading to neuronal cell loss^1,2^. However, the TAU protein in the brain of AD patients has recently been shown to be abnormally hyper-phosphorylated, which is better associated with cognitive dysfunction than accumulation of amyloid^3^. In addition, increasing evidence also suggests that Ca^2+^ signaling dysregulation mediates accumulation of the unfolded proteins, which triggers endoplasmic reticulum (ER) stress, in turn resulting in synaptic dysfunction in AD^4–7^. However, it is unclear how Ca^2+^ dysregulation is involved in ER stress and Aβ in neuronal cells.

One of the Ca^2+^ regulatory proteins, ryanodine receptor (RyR2) is a huge tetrameric protein that mediates Ca^2+^ release from sarco(endo)plasmic reticulum^8^. To date, more than 150 mutations within RyR2 have been linked with catecholaminergic polymorphic ventricular tachycardia (CPVT). The point mutations are not randomly distributed, but cluster in three “hot” domains: i.e. the N-terminal domain (aa 1–600), the central domain (aa 2000–2500), and the C-terminal domain^9,10^. We have previously reported that either a single point mutation, hyperphosphorylation, or oxidative stress commonly causes domain unzipping between the N-terminal and central domains, which allosterically displaces calmodulin (CaM) from RyR2, thus leading to Ca^2+^ leakage in a CPVT-associated *RyR2*^*R2474S/*+^ knock-in mouse model^9–12^ and also in a canine model of tachycardia-induced heart failure^13–17^. Conversely, dantrolene, a specific drug for treatment of malignant hyperthermia, which binds to the RyR2 N-terminal domain (aa 601–620), stabilized the domain-domain interactions from the unzipped to the zipped state, and hence restored normal CaM binding to RyR2, thereby inhibiting Ca^2+^ leakage in either CPVT-associated *RyR2*^*R2474S/*+^ mouse model^9^, tachycardia-induced canine heart failure model^15^, or pressure-overload induced mouse heart failure model^18^.

Dantrolene has also been shown to ameliorate Ca^2+^ handling in RyR2 on the ER membrane in neuronal cells, preventing accumulation of amyloid in the hippocampus^19^. However, the molecular mechanism involved in the beneficial effect of dantrolene is unclear. Based on the fact that dantrolene can inhibit Ca^2+^ leakage by inducing association of CaM to cardiac RyR2, we hypothesized that Ca^2+^ leakage from ER is also induced by a variety of stresses, thereby causing structural or functional aberrations in neuronal cells; for example, Ca^2+^ leakage caused by dissociation of CaM from RyR2 may accelerate accumulation of unfolded proteins in ER, leading to ER stress. ER stress has been shown to activate the unfolded protein response (UPR), which inhibits protein synthesis and promotes proper folding of various proteins^20^. When UPR does not function properly or reaches a maximum level, protein degradation is favored leading eventually to apoptosis. Therefore, we hypothesized that blocking Ca^2+^ leakage from RyR2 in neuronal cells may lead to decreased ER stress.

Recently, we found that the V3599K substitution – out of 25 substitutions – in the CaM-binding domain in RyR2 showed the highest binding affinity for CaM. Hence, we generated a *RyR2*^*V3599K/*+^ mouse model with a single amino acid substitution in the CaM-binding domain of RyR2, and crossed it to the CPVT–associated *RyR2*^*R2474S/*+^ mouse to obtain an *RyR2*^*R2474S/*+*/V3599K/*+^ mouse. Interestingly, All CPVT phenotypes—bidirectional or polymorphic ventricular tachycardia, spontaneous Ca^2+^ transients, and Ca^2+^ sparks—were reversed in *RyR2*^*R2474S/*+*/V3599K/*+^ mice^21^. By using the *RyR2*^*V3599K/V3599K*^ mice, it may be possible to determine the critical role of RyR2-bound CaM in the pathogenesis of AD.

The aim of this study was to clarify whether dissociation of CaM from RyR2 and subsequent Ca^2+^ leakage is involved in the pathogenesis of AD, and if so, whether increasing the affinity of CaM to RyR2 can prevent AD development.

## Results

*App*^*NL-G-F/NL-G-F*^ mice (*App*^*NL-G-F*^ mice), which harbor three familial AD mutations (Swedish: KM670/671NL, Beyreuther/Iberian: I716F, and Arctic: E693G) in the amyloid-β precursor protein, show cognitive dysfunction and accumulation of Aβ within several months^22^. We investigated whether enhancement of CaM binding to RyR2 influences development of AD, using *App*^*NL-G-F*^ mice and *RyR2*^*V3599K/V3599K*^ mice (*RyR2*^*V3599K*^). *RyR2*^*V3599K*^ mice showed no apparent abnormalities. To examine whether stabilization of RyR2 through enhanced CaM binding is critical for AD pathogenesis, we generated *App*^*NL-G-F*^/*RyR2*^*V3599K*^ double homozygous mice (*App*^*NL-G-F*^/*RyR2*^*V3599K*^ mice), and evaluated how the AD phenotype is modified (Supplementary Fig. 1).

**Figure 1:**
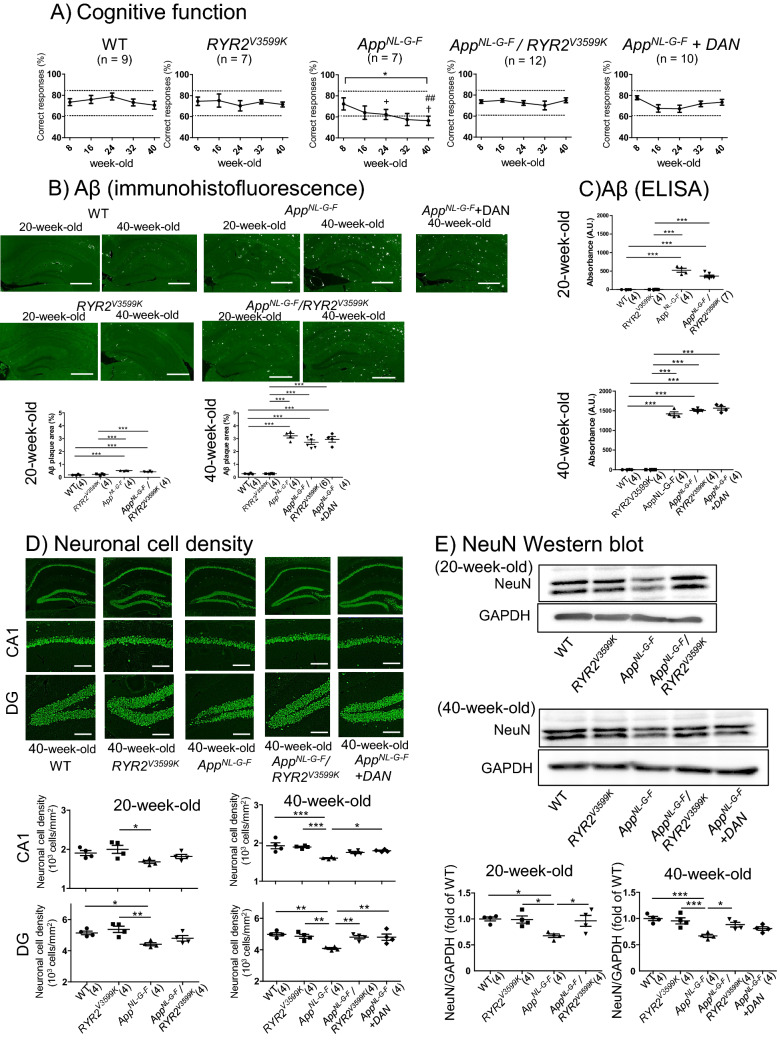
Long-term time course of spatial working memory, Aβ, and number of neuronal cells in WT, *App*^*NL-G-F*^, *App*^*NL-G-F*^ + dantrolene (DAN), *RYR2*^*V3599K*^, and *App*^*NL-G-F*^/*RYR2*^*V3599K*^ mice. (**A**) Time course of spatial working memory as aging. The Y-maze test was used for the evaluation of spatial working memory. Values for individual mice are plotted as mean ± SEM. N = 7–12 mice. Dotted line indicates standard deviation of 8-week-old WT mice. *p < 0.05 versus 8-week-old *App*^*NL-G-F*^ mice (two-way ANOVA with post-hoc Dunnett test). + *p* < 0.05 vs. 24-week-old WT mice, ^†^*p* < 0.05 versus 40 week-WT and -*RYR2*^*V3599K*^ mice, ##*p* < 0.01 vs. 40 week-old-*App*^*NL-G-F*^/*RYR2*^*V3599K*^ and -*App*^*NL-G-F*^ + dantrolene (DAN) mice (one-way ANOVA with post-hoc Tukey’s multiple comparison test). (**B**) Amyloid beta (Aβ) in a cross section of hippocampus and the summarized data. Aβ was evaluated by immunocytochemistry using anti-Aβ. Aβ was expressed as an area (%) normalized by hippocampus area. Scale bars: 500 μm. N = 4–6 mice. Parentheses indicate the number of mice. The cross sections of whole brains are presented in Supplementary Fig. 2. ***p < 0.001 (one-way ANOVA with post-hoc Tukey’s multiple comparison test). (**C**) The summarized data of Aβ evaluated by ELISA. each datum point reveals the average value of Aβ obtained form 20-week and 40-week mouse brains. N = 4–7 mice. Parentheses indicate the number of mice. ***p < 0.001 (one-way ANOVA with post-hoc Tukey’s multiple comparison test). (**D**) NeuN stained cells and the summarized data. Neuronal cell density in a slice of Cornu Ammonis (CA) 1 and the dentate gyrus (DG) of the hippocampus (3 μm thickness) were measured by counting all NeuN stained cells devided CA 1 or DG area, and values for individual mice are plotted together with mean ± SEM. Scale bars: 100 μm. Parentheses indicate the number of mice. *p < 0.05, **p < 0.01, ***p < 0.001. (one-way ANOVA with post-hoc Tukey’s multiple comparison test). E) Representative Western blots for NeuN in the isolated hippocampus, and the summarized data. Each datum point reveals the average value of NeuN obtained from 20-week and 40-week mouse brains. N = 4 mice. Parentheses indicate the number of mice. Full-length blots are presented in Supplementary Fig. 10. *p < 0.05, ***p < 0.001 (one-way ANOVA with post-hoc Tukey’s multiple comparison test).

Consistent with a previous report^22^, spatial working memory was severely inhibited (Fig. 1A), with significant accumulation of Aβ in *App*^*NL-G-F*^ mice (Fig. 1B,C, Supplementary Fig. 2). Interestingly, cognitive dysfunction was restored at 20 and 40 weeks in *App*^*NL-G-F*^/RyR2^V3599K^ mice, and at 40 weeks in dantrolene-treated *App*^*NL-G-F*^ mice (Fig. 1A). There was hardly any accumulation of Aβ in the neural tissue until 40 weeks in WT and *RyR2*^*V3599K*^ mice, whereas Aβ markedly accumulated in *App*^*NL-G-F*^, dantrolene-treated *App*^*NL-G-F*^, and *App*^*NL-G-F*^/*RyR2*^*V3599K*^ mice. There was no significant difference in Aβ levels at 20 and 40 weeks between *App*^*NL-G-F*^, dantrolene-treated *App*^*NL-G-F*^, and *App*^*NL-G-F*^/*RYR2*^*V3599K*^ mice (Fig. 1B,C). Of particular interest, significant neuronal cell loss occurred in *App*^*NL-G-F*^ mice, whereas it did not occur in dantrolene-treated *App*^*NL-G-F*^ and *App*^*NL-G-F*^/*RYR2*^*V3599K*^ mice (Fig. 1D). The neuronal cell loss was also confirmed by a decrease in NeuN in the isolated hippocampus of *App*^*NL-G-F*^, which was rescued by genetic gene substitution of RyR2 V3599K. Dantrolene also tended to rescue the neuronal cell loss in 40-week-old *App*^*NL-G-F*^ mice (Fig. 1E).

**Figure 2:**
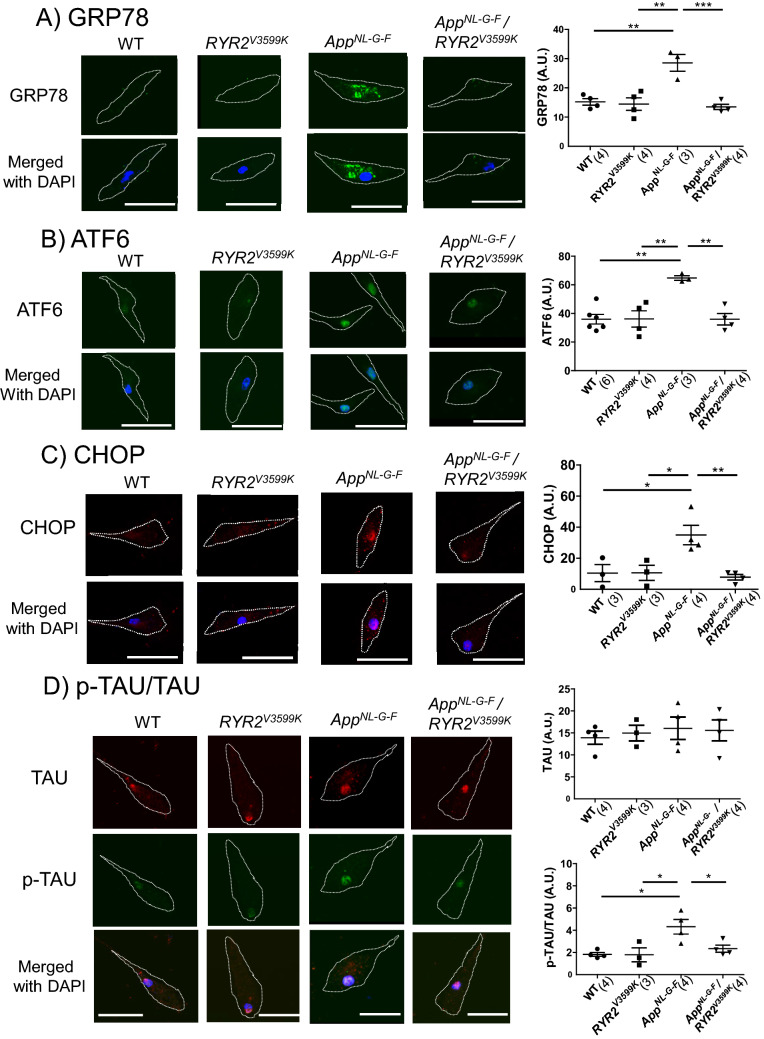
Protein expression of ER stress markers, TAU and p-TAU in WT, *App*^*NL-G-F*^, *RYR2*^*V3599K*^, and *App*^*NL-G-F*^/*RYR2*^*V3599K*^ incubated neuronal cells. Representative images of (**A**) GRP78, (**B**) ATF6, (**C**) CHOP, (**D**) TAU/p-TAU obtained from single cells, and the summarized data. Merged images with DAPI staining are also shown. p-TAU was expressed as the ratio to total TAU. Cell shapes are indicated by dotted lines. Values for individual mice are plotted together with mean ± SEM. Scale bars: 30 μm. N = 20–33 cells from 3 to 6 mice. Parentheses indicate the number of mice. *p < 0.05, **p < 0.01, ***p < 0.001 (one-way ANOVA with post-hoc Tukey’s multiple comparison test).

Next, we examined the protein expression of Glucose-Regulated Protein 78kD (GRP78), TAU, and the phosphorylation level of TAU (p-TAU) in whole brain homogenate. As shown in Supplementary Fig. 3, there was no significant difference in GRP78, TAU, p-TAU between all groups. These data are consistent with previously reported findings that no endoplasmic reticulum stress response was observed when using the same *App*^*NL-G-F*^ mice^23^. So, we investigated the possibility that changes of ER stress markers and pTAU/TAU are limited to the neuronal cells. We examined the localization and protein expression of ER stress markers {GRP78, Activating Transcription Factor 6 (ATF6), C/EBP-homologous protein (CHOP)} and p-TAU/TAU in cultured neuronal cells at 20 weeks of age. Expressions of GRP78, ATF6 and CHOP increased in *App*^*NL-G-F*^ neuronal cells compared to WT and *RYR2*^*V3599K*^ neuronal cells, but these expressions were restored to normal in App^NL-G-F^ / RYR2^V3599K^ neuronal cells (Fig. 2A–C). In *App*^*NL-G-F*^ neuronal cells, GRP78 was expressed in the cytosol, but ATF6 and CHOP were expressed in the nucleus (Fig. 2A–C). There was no difference in the expression level of TAU between WT, *App*^*NL-G-F*^, *RYR2*^*V3599K*^, and *App*^*NL-G-F*^/*RYR2*^*V3599K*^ neuronal cells. However, the phosphorylation level of TAU (p-TAU) increased dramatically in *App*^*NL-G-F*^ neuronal cells compared to neuronal cells from the other mouse strains (Fig. 2D).

**Figure 3:**
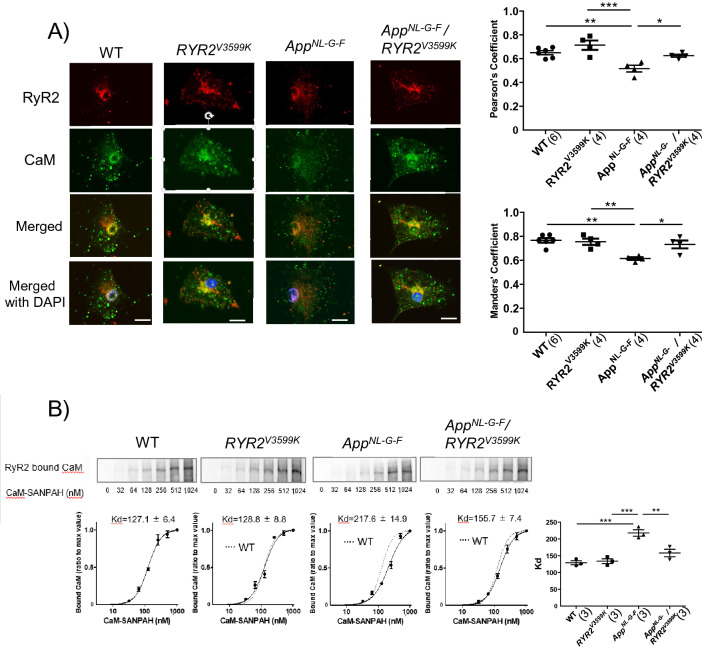
CaM-RyR2 interaction in WT, *App*^*NL-G-F*^, *RYR2*^*V3599K*^, and *App*^*NL-G-F*^/*RYR2*^*V3599K*^ mice. (**A**) Representative images of endogenous CaM, and RyR2 in neuronal cells.; (Left) immuno-staining of RyR2 (red), immuno-staining of CaM (green) and merged image (Right) the summarized data of the Pearson’s and Manders’ coefficients. Scale bars: 10 μM. Values for individual mice are plotted together with mean ± SEM. N = 25–48 cells from 4–6 mice. Parentheses indicate the number of mice. *p < 0.05, **p < 0.01 (one-way ANOVA with post-hoc Tukey’s multiple comparison test). (**B**) (Top) Representative immunoblots of the RyR2-bound CaM–SANPAH (a photoreactive crosslinker; N-succinimidyl-6-[4′-azido-2′-nitrophenylamino]). (Bottom) Summarized data of CaM binding to RyR2 as a function of the concentration of CaM–SANPAH. CaM binding was expressed as the ratio to the maximum binding at 1024 nM CaM. Dotted line means CaM binding curve in WT. Data represent means ± SEM of 3 mice. Parentheses indicate the number of mice. Full-length blots are presented in Supplementary Fig. 11. **p < 0.01, ***p < 0.001 (one-way ANOVA with post-hoc Tukey’s multiple comparison test).

At 20 weeks of age when cognitive dysfunction occurred, which is associated with Aβ accumulation and TAU phosphorylation in *App*^*NL-G-F*^ mice, we measured the localization and expression level of RyR2 and CaM in isolated neuronal cells. CaM was largely co-localized with RyR2 especially around the nucleus where ER is known to be enriched (Fig. 3A). In *App*^*NL-G-F*^ neuronal cells, CaM around the nucleus was dissociated from RyR2, whereas co-localization was restored in *App*^*NL-G-F*^/*RYR2*^*V3599K*^ neuronal cells (Fig. 3A). Direct association of exogenous CaM with RyR2 was also evaluated by binding of exogenous CaM cross-linked with sulfosuccinimidyl-6-[4′-azido-2′-nitrophenylamino]hexanoate (Sulfo-SANPAH) to RyR2^11^. The apparent binding affinity of exogenous CaM to RyR2 decreased in *App*^*NL-G-F*^ neural tissue, whereas it was restored in *App*^*NL-G-F*^/*RYR2*^*V3599K*^ neural tissue (Fig. 3B). The finding that cognitive dysfunction was ameliorated along with attenuation of TAU phosphorylation, but without decrease in Aβ in APP^NL-G-F^/*RYR2*^*V3599K*^ mice, strongly suggests that CaM dissociation from RyR2 play a critical role in cognitive dysfunction during AD development. On the other hand, at 8,20 and 40 W, there was no significant difference in RyR2 expression in brain homogenates between all groups (Supplementary Fig. 4). We also measured the changes in intracellular Ca^2+^ before and after addition of ionomycin (10 μM) to neuronal cells isolated from WT, RyR2^V3599K^, App^NL-G-F^, and App^NL-G-F/RYR2V3599K^ mice brain tissue at 20 weeks of age (Supplementary Fig.5). The time to peak was longer in App^NL-G-F^ neuronal cells, suggesting a decrease in ER Ca^2+^ content in App^NL-G-F^ neuronal cells.

**Figure 4:**
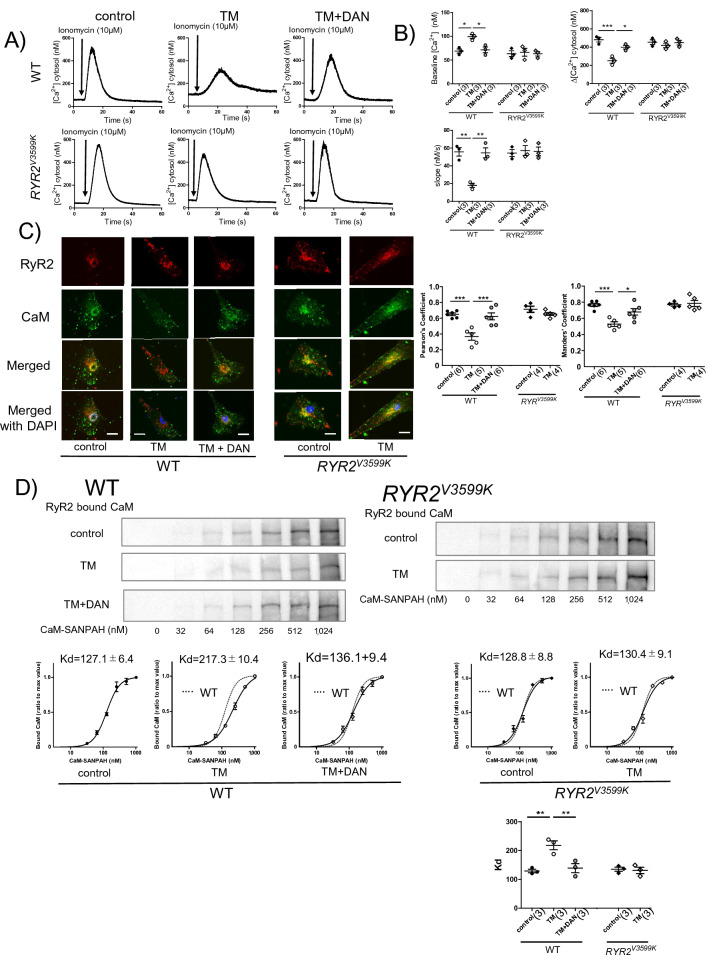
Effect of tunicamycin (TM) and dantrolene (DAN) on Ca^2+^ release characteristics and CaM-RyR2 interaction in WT and *RYR2*^*V3599K*^ neuronal cells. (**A**) Representative images of intracellular Ca^2+^ before and after addition of ionomycin (10 μM). (**B**) Summarized data of baseline Ca^2+^, increased cytosolic [Ca^2+^] after addition of ionomycin (Δ[Ca^2+^]), rate of release (slope). Values for individual mice are plotted together with mean ± SEM. N = 25–34 cells from 3 mice. Parentheses indicate the number of mice. *p < 0.05, **p < 0.01, ***p < 0.001 (one-way ANOVA with post-hoc Tukey’s multiple comparison test). (**C**) Representative images of endogenous CaM and RyR2 in neuronal cells. Multiple experiments were performed simultaneously with a single control experiment; (Left) immuno-staining of RyR2 (red), immuno-staining of CaM (green) and merged image. (Right) the summarized data of the Pearson’s and Manders’ coefficients. Scale bars: 10 μm. Values for individual mice are plotted together with mean ± SEM. N = 21–27 cells from 4–6 mice. Parentheses indicate the number of mice. *p < 0.05, ***p < 0.001 (one-way ANOVA with post-hoc Tukey’s multiple comparison test in WT, unpaired *t* test in *RYR2*^*V3599K*^). (**D**) Representative immunoblots of the RyR2-bound CaM–SANPAH (a photoreactive crosslinker; N-succinimidyl-6-[4′-azido-2′-nitrophenylamino]). (Bottom) Summarized data of CaM binding to RyR2 as a function of the concentration of CaM–SANPAH. The CaM binding was expressed as the ratio to the maximum binding at 1,024 nM CaM. Dotted line means CaM binding curve in WT. Data represent means ± SEM of 3 mice. Parentheses indicate the number of mice. Full-length blots are presented in Supplementary Fig. 12 **p < 0.01 (one-way ANOVA with post-hoc Tukey’s multiple comparison test in WT, unpaired *t* test in *RYR2*^*V3599K*^).

**Figure 5:**
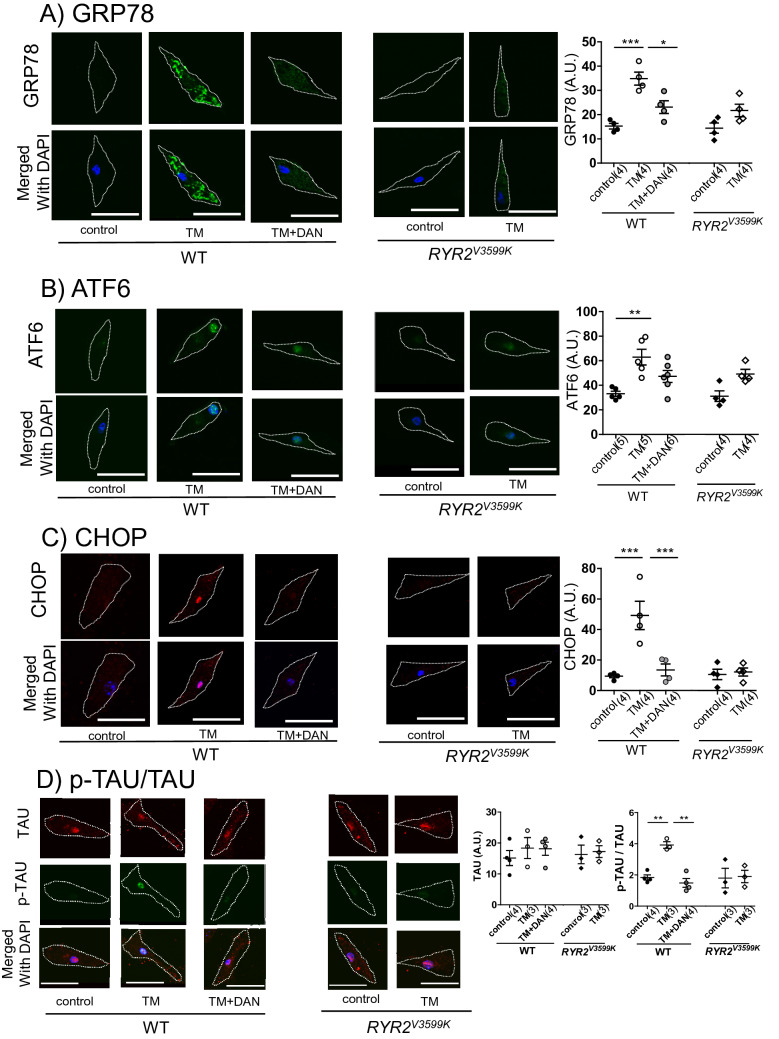
Change in protein expression level of ER stress marker after addition of TM to WT and *RYR2*^*V3599K*^ neuronal cells. Representative images of (**A**) GRP78, (**B**) ATF6, (**C**) CHOP, (**D**) TAU/p-TAU, and the summarized data. Merged images with DAPI staining were also shown. p-TAU was expressed as the ratio to total TAU. DAN: dantrolene. Scale bars: 30 μm. Values for individual mice are plotted together with mean ± SEM. N = 19–32 cells from 3 to 6 mice. Parentheses indicate the number of mice. *p < 0.05, **p < 0.01, ***p < 0.001 (one-way ANOVA with post-hoc Tukey’s multiple comparison test in WT, unpaired *t* test in *RYR2*^*V3599K*^).

To further investigate how destabilization of RyR2 is linked with TAU phosphorylation and ER stress in neuronal cells, we examined the effect of tunicamycin (TM), a well-known ER stress inducer^24,25^, on baseline cytosolic [Ca^2+^], response to ionomycin (10 μM), TAU phosphorylation, and ER stress in isolated neuronal cells. After addition of TM (3 μM) for 50 min, baseline cytosolic [Ca^2+^] increased in WT neuronal cells, but not in the presence of dantrolene. Of interest, baseline cytosolic [Ca^2+^] was not elevated after adding TM in *RYR2*^*V3599K*^ neuronal cells (Fig. 4A,B). Furthermore, in the presence of TM, ionomycin reduced the peak cytosolic [Ca^2+^] and slowed the rate of increase (slope) and the amount of increase in cytosolic Ca^2+^ (Δ[Ca^2+^]), suggesting a decrease in ER Ca^2+^ content in WT neuronal cells. However, this was not the case with WT neuronal cells treated with dantrolene or *RYR2*^*V3599K*^ neuronal cells.

The amount of CaM co-localized with RyR2 decreased after addition of TM in WT neuronal cells, whereas co-localization was restored in dantrolene-treated neuronal cells and in *RYR2*^*V3599K*^ neuronal cells (Fig. 4C). The apparent binding affinity of exogenous CaM to RyR2 also decreased after addition of TM in WT neuronal tissue, but not in dantrolene-treated neuronal tissue and *RYR2*^*V3599K*^ neuronal tissue (Fig. 4D).

Since decrease in ER Ca^2+^ content has been reported to increase ER stress, which inhibits clearance of misfolded proteins^26^, we examined the effect of TM on localization and protein expression of GRP78, ATF6, and CHOP in isolated neuronal cells. GRP78, ATF6 and CHOP all increased 24 h after addition of TM in WT neuronal cells, whereas these changes were not observed in dantrolene-treated WT neuronal cells and *RYR2*^*V3599K*^ neuronal cells (Fig. 5A–C). GRP78 was expressed in the cytosol after addition of TM in WT neuronal cells, while both ATF6 and CHOP were expressed in the nucleus. p-TAU increased after addition of TM in WT neuronal cells, but not in dantrolene-treated WT neuronal cells and in *RYR2*^*V3599K*^ neuronal cells (Fig. 5D). These findings are consistent with our results in *App*^*NL-G-F*^ neuronal cells. Since TM has been previously shown to diminish the antioxidant capacity via inhibition of reduced glutathione (GSH) synthesis in liver cells^25^, we assessed whether TM may increase oxidative stress level in isolated neuronal cells (Supplementary Fig. 6). Reactive oxygen species (ROS), evaluated by DCFH-DA fluorescence, increased similarly after addition of TM in both WT and *RYR2*^*V3599K*^ neuronal cells, and dantrolene did not reduce ROS, suggesting that oxidative stress is upstream from CaM dissociation from RyR2 (Supplementary Fig. 6). In support of this notion, we previously reported that oxidative stress directly induces Ca^2+^ leakage from cardiac RyR2 owing to domain unzipping between the N-terminal and central domains and subsequent CaM dissociation from RyR2^27,28^.

TM inhibits the DPAGT1 enzyme to inhibit one of the first steps of glycoprotein biosynthesis in the ER which results in the accumulation of misfolded proteins to cause subsequent ER stress^29^. Therefore, a mechanism that increases ER stress via DPAGT1 is also conceivable, although it is not known whether DPAGT1 dysfunction is involved in abnormal Ca^2+^ processing in neurons.

We then evaluated whether chronic accumulation of Aβ increases ROS in isolated neuronal cells. Compared to WT or *RyR2*^*V3599K*^ mice, ROS markedly increased in *App*^*NL-G-F*^ mice (Supplementary Fig. 7). Of interest, ROS increased similarly in *App*^*NL-G-F*^/*RYR2*^*V3599K*^ mice as well, again suggesting that the increase in ROS is more upstream than Ca^2+^ leakage during AD development (Supplementary Fig. 7).

Collectively, we showed that accumulation of Aβ in genetic mouse models leads first in increased oxidative stress, then in dissociation of CaM from RyR2, in Ca^2+^ leakage from ER, and in decreased ER Ca^2+^ content, which promotes ER stress response, along with TAU phosphorylation. Pharmacological (by dantrolene) or genetic enhancement of CaM binding to RyR2 (by V3599K substitution in RyR2) inhibits Ca^2+^ leakage, thereby restoring ER Ca^2+^ content, and decreasing ER stress.

## Discussion

In this study, we found that RyR2 destabilization due to CaM dissociation plays an essential role in the decrease in ER Ca^2+^ content, increase in ER stress and p-TAU, subsequently leading to neuronal cell loss and cognitive dysfunction in *App*^*NL-G-F*^ mice, and that either genetic (by V3599K substitution in RyR2) or pharmacological (by dantrolene) inhibition of CaM dissociation from RyR2 protected from AD (Supplementary Fig. 8).

The amyloid cascade hypothesis has been widely recognized as a possible pathogenic mechanism of AD^2^. Currently, much effort has been made to reduce amyloid production or eliminate amyloid from the neural tissue. Unfortunately, most promising drugs failed to show any potential benefit for patients in clinical trials^5^, due to multiple reasons. First, accumulation of Aβ in neuronal cells may be only one of the possible factors that affect the pathogenesis of AD^2^. Second, inhibition of secretase activity to restrict toxic amyloid production may be harmful due to the many substrates that these enzymes target thus causing pleiotropic effects. For instance, γ-secretase modulates Notch signaling that plays a critical role in cell differentiation^30^. The fact that both neuronal cell loss and cognitive dysfunction were ameliorated without decreasing Aβ in chronically dantrolene-treated *App*^*NL-G-F*^ mice and in *RYR2*^*V3599K*^ mice clearly indicates that accumulation of Aβ triggers initiation of an abnormal process leading to AD, but it is not necessary and sufficient for the pathogenesis of AD. Hence obviously other mechanisms need to be considered, particularly regarding AD treatment.

Defective Ca^2+^ homeostasis is another plausible mechanism involved in the pathogenesis of AD^5,6^. In support of this hypothesis, it has been reported that amyloid accumulation is linked with elevation of cytosolic Ca^2+^ in neurons^31^, and that dysregulated ER Ca^2+^ release alters synaptic transmission and plasticity mechanisms in an AD-type mouse model before the onset of histopathology and cognitive deficits^32^. Our study strongly suggests that displacement of CaM from RyR2 and subsequent ER Ca^2+^ leakage increases ER stress in neuronal cells, thereby causing neuronal cell loss and cognitive dysfunction. Of interest, leaked Ca^2+^ and displaced CaM induce activation of both Ca^2+^ /calmodulin-dependent protein kinase II (CaMKII) and calcineurin (CaN) signals, both of which play important roles in the regulation of cognitive function^33,34^. CaN activates additional phosphatases, such as protein phosphatase 1, which further induces long-term depression that erases memories^35^. CaMKII has also been suggested to be a TAU kinase^36^. Hyper-activation of CaMKII may increase TAU phosphorylation; therefore, CaMKII is implicated in AD pathogenesis. Activation of CaN by CaM also disrupts the phosphatases interaction with TAU, possibly leading to TAU hyperphosphorylation^37^. Moreover, it has been recently shown that dantrolene improved cognitive dysfunction in association with decreased accumulation of amyloid^38–41^, thus linking defective Ca^2+^ homeostasis with AD, although the precise mechanism has not been clarified yet.

In the cardiac muscle tissue, we previously reported that dantrolene specifically binds to amino acids 601–620 of RyR2 (corresponding with amino acids 590–609 in RyR1 to which dantrolene has been reported to bind^42^), and restores defective N-terminal and central domain-domain interactions (domain unzipping), prevents dissociation of CaM from RyR2, suppresses aberrant Ca^2+^ release, and thus inhibits lethal arrhythmia^43^ and progression of heart failure^15^. Since RyR2 is abundantly expressed in the neural tissue, we anticipated that dantrolene also contributes to stabilization of RyR2 in AD-type diseased neural tissue. As expected, we observed that dantrolene inhibited ER Ca^2+^ depletion by enhancing CaM binding affinity to RyR2 in TM-induced AD-type cells, thereby reducing ER stress and TAU phosphorylation. In contrast to the aforementioned reports, including ours, that showed a beneficial effect of dantrolene on AD prevention, one report showed that dantrolene rather promoted cognitive dysfunction, associated with further accumulation of amyloid^44^. There is no clear reason to account for the contradictory findings between these studies, although the experimental models used, the dosage, route, period, or timing of dantrolene administration can be speculated as influential factors. In our study, we did not observe any abnormal accumulation of Aβ or harmful effect after administration of dantrolene, although mice were fed with more than 20 times higher dose (100 mg/Kg/day) than the mice in Zhang, et al.’ s study (5 mg/kg/day p.o., twice a week).

Although the effect of dantrolene on RyR2 channel function is specific^15^, we cannot entirely exclude possible off-target effects of dantrolene. In order to determine the critical role of RyR2 dysregulation in the pathogenesis of AD, a genetic approach is essential. In this study, we clearly demonstrated that when binding of CaM to RyR2 was stabilized pharmacologically in TM-induced AD-type cells or genetically in neural cells from *App*^*NL-G-F*^ mice, most AD-related phenotypes (i.e. ER stress, neuronal cell loss, and cognitive dysfunction), except for Aβ accumulation were rescued. Recently TAU phosphorylation, rather than amyloid accumulation, has been recognized as an important determinant in the pathogenesis of developing AD^45^. Our finding that TAU phosphorylation, but not Aβ accumulation, was associated with improvement of cognitive function supports this idea. However, the fact that stabilization of RyR2 through increased binding affinity to CaM almost completely inhibited TAU phosphorylation in either TM-induced AD-type cells or neural cells from *App*^*NL-G-F*^ mice strongly suggests that destabilization of RyR2 is an upstream of TAU phosphorylation in the pathogenesis of AD (Supplementary Fig. 8).

The mechanism by which the V3599K mutation prevents the decrease in CaM binding affinity for RyR2 in *App*^*NL-G-F*^ mice remains unclear. Since the RyR2 V3599K mutation enhances the binding affinity of CaM to RyR2 in *App*^*NL-G-F*^ mice or only when WT mice is subjected to TM (Figs. 3B, 4D), the accessibility of CaM near the V3599K mutation site of RyR2 can be allosterically enhanced only if there is a defective inter-subunit interaction in the tetrameric structure of RyR2. Recent higher-order structural analysis of RyR2 supports this idea. Namely, since CaM interacts closely with three domains of RyR2 (helix α − 1, helix 2b, helix α − 9)^46^, the accessibility of in situ CaM to its binding site may not be easily enhanced by the V3599K mutation. In contrast, the defective inter-subunit interaction due to ER stress may allosterically change the conformational state of the CaM binding region, facilitating CaM binding to the V3599K mutant (not WT) domain in helix α − 1. Of interest, dantrolene binds to a specific site (601–620 a.a.) near both the zipping interface and CaM, supporting the idea that dantrolene prevents CaM dissociation by stabilizing inter-subunit interaction (Supplementary Fig. 8).

There are several limitations in this study. First, the isolation process can affect the function of neuronal cells differently depending on the disease of each mouse model. Second, it is not clear whether the dissociation of CaM from RyR2 induced by endoplasmic reticulum stress preferentially targets neurons or also affects astrocyte / glial / non-neuronal cell types.

In conclusion, CaM dissociation from RyR2 plays a crucial role in the pathogenesis of AD, and enhancing CaM binding to RyR2 may be a novel, potent therapeutic strategy against AD. Especially, the discovery that neuronal cell loss can be fully prevented simply by stabilizing RyR2 would shed new light on the treatment of AD.

## Methods

### Chemicals

Dantrolene and ionomycin were purchased from Fuji Film Wako Chemicals, Tokyo, Japan. L-polyornithine was purchased from Sigma Aldrich, St Louis, USA. Sulfo-SANPAH was purchased from Thermo Fisher Scientific, Waltham, USA.

### Animals

WT C57BL/6 mice were obtained from Japan SLC, Inc. (Hamamatsu, Japan). *RYR2*^*V3599K*^ KI mice were made by UNITECH Co., Ltd (Chiba, Japan). *App*^*NL-G-F*^ mice were from RIKEN institute. All in vivo experiments were performed in contemporary random order. All strains were maintained on a C57BL/6 background. Measurement and analysis of the acquired data were performed by the investigators without knowing any genetic information of the mice.

### Dantrolene administration

Dantrolene-treated *App*^*NL-G-F*^ mice were fed, using a feeding apparatus (Roden CAFÉ; Oriental Yeast Co., Ltd., Tokyo, Japan), with 100 mg/kg/day dantrolene (Fuji Film Wako Chemicals, Tokyo, Japan) for 36 weeks (from 4-week-old to 40-week-old). The oral dose of dantrolene for chronic administration was determined as the dose by which inducible ventricular tachycardia was almost completely inhibited in CPVT-type *RyR2R2474S*+*/−* knock-in mice^9,47^. To avoid excessive moisture of dantrolene, food including dantrolene was changed at least every three days. Body weight was monitored during chronic administration of dantrolene; in all mice, body weight increased similarly regardless of dantrolene administration. The weight of the food consumed by every mouse was monitored and the concentration of dantrolene mixed in the food was adjusted at 100 mg/kg/day. In most cases, concentration of dantrolene in the food was 0.25%. No animals exhibited signs of toxicity and no mortality was detected in any group.

### Y-maze test

The Y-maze test was used for the evaluation of spatial working memory, at 8-, 16-, 24-, 32-, and 40-week-old. The Y-maze consisted of three arms (each 50 cm length, 10 cm width, and 15 cm height). The mouse was placed in the center of the Y-maze and allowed to move freely for 8 min. The coefficient of alternation (CA) was calculated as follows: CA = Nright/Ntotal, where Nright: the number of right entries into a new arm and Ntotal: the total number of entries.

### Buffers and materials

Preparation of buffers and materials were as described by Eide et al.^48^. Twenty-five milligrams papain (Worthington Biochemical, Lakewood, NJ, USA) was dissolved in HEPES-buffered saline (10 mM HEPES, 145 mM NaCl, 22 mM KCl, 5 mM glucose, pH 7.3) to 2 mg/mL and kept at 4 °C. Plating media comprised of Dulbecco's modified Eagle medium (DMEM) supplemented with high glucose (Invitrogen, Carlsbad, CA, USA), 10% fetal calf serum (FBS), 10% F-12 (Invitrogen), penicillin (100 U/mL), and streptomycin (100 µg/mL). Feeding media comprised B27 neurobasal media (Invitrogen) supplemented with penicillin (100 U/mL) and streptomycin (100 µg/mL).

### Polyornithine coating of culture dishes

Plastic culture dishes were coated with l-polyornithine prior to use as described by Eide et al.^48^. Each 35-mm plate was incubated overnight in 2.0 mL (or 0.5 mL for 24-dish plate) of 0.5 mg/mL borate buffer (10 mM Na2B4O7, pH 8.4). After incubation, plates were washes twice with water.

### Isolation of neurons

Primary neuronal cultures were obtained from 20-week-old mice as described by Eide et al.^48^. Mice were anesthetized with overdose pentobarbital. The whole brain was isolated under sterile conditions, rinsed in HBS, and minced into small pieces. The minced tissue (approximately 0.5 mL) was then treated with 1 mL papain for 15 min at 37 °C. After gently leaving the solution, the papain/HBS supernatant was removed and replaced with 12 mL of prewarmed plating medium (2.0 mL/35-mm dishes), after which the tissue was immediately triturated. Approximately 2 mL of this cell solution was added to each 35-mm L-polyornithine-coated dish. Cells were allowed to settle for 15 min, washed once, and replaced with prewarmed plating medium (2.0 mL/35-mm dish). After plating, cells were incubated at 37 °C in 5% CO2 and incubated for 7 days without media replacement. On day 7, the plating media was replaced with the same volume of feeding medium and used for experiments.

### Monitoring of intracellular reactive oxygen species (ROS) in cultured neurons

In mouse neuronal cells, a fluorescent probe, 2′,7′-dichlorofluorescin diacetate (DCFH-DA, Thermo Fisher Scientific, Waltham, USA), was used for the assessment of intracellular reactive oxygen species (ROS) formation^49^. This assay is widely used as a reliable method for the measurement of intracellular ROS such as hydrogen peroxide (H2O2), hydroxyl radicals (OH·), and hydroperoxides (ROOH). Fluorescent images (excitation at 490 nm and emission at 530 nm) were acquired with a microscope (LSM 510 Carl Zeiss).

### Monitoring of intracellular Ca^2+^ in cultured neurons

Cal520 (Kd = 320 nM) was used as a fluorescent Ca^2+^ probe to detect cytoplasmic calcium. Isolated neurons were incubated with 1 μM Cal520 AM for 50 min in a 37 °C incubator and washed twice with Tyrode's solution without calcium (140 mM NaCl, 6 mM KCl, 2 mM CaCl_2_, 1 mM MgCl_2_, 10 mM glucose, and 5 mM HEPES-Tris, pH 7.4). Intracellular Ca^2+^ was measured using a fluorescent digital microscope (BZ9000, Keyence, Japan, Plan 20 × Objective, Nikon, Japan). ER Ca^2+^ content was measured as an increase in Ca^2+^ fragments mobilized from ER store induced by 10 μM ionomycin. Then, fluorescence of Fmax and Fmin were measured by addition of Ca^2+^ (1 mM [Ca^2+^]) and 20 mM BAPTA (1,2- b is (o—a mino p henoxy) ethane—N, N, N′, N′—tetra acetic acid), respectively (Supplementary Fig. 9). The absolute concentration of Ca^2+^ was calculated by the following formula.

$$ {\text{Ca}}^{{{2} + }} = {\text{Kd }}\left( {{\text{F}}{-}{\text{Fmin}}} \right)/\left( {{\text{Fmax}}{-}{\text{F}}} \right);\quad {\text{Kd}} = {32}0\,{\text{nM}} $$

### Immunocytochemistry analysis

Antibodies against the following proteins were used for immunohistochemistry: RyR2 (C3-33 1/250, Sigma-Aldrich, St. Louis, USA), CaM (EP799Y 1/250, Abcam, Cambridge, UK), TAU (TAU-5 1/100, Abcam, Cambridge, UK), p-TAU (EPR2731 1/500, Abcam, Cambridge, UK), GRP78 (PA5-19503 1/600, Thermo Fisher Scientific, Waltham, USA), CHOP (9C8 1/50, Santa Cruz Biotechnology, Dallas, USA), and ATF6 (PA5-72554 1/250, Thermo Fisher Scientific, Waltham, USA). Cultured neuronal cells were fixed with 4% paraformaldehyde in PBS for 5 min, washed three times with PBS, and permeabilized in 0.5% Triton X-100 and 1% BSA for 20 min. Then, the neuronal cells were incubated overnight at 4 °C with the first antibodies in 1% BSA and 0.5% Triton X-100, followed by labeling with an Alexa 488-conjugated secondary antibody (1/300, for ATF6, GRP78, p-TAU, CaM), Alexa 633-conjugated secondary antibody (1/300, for RyR2, CHOP, TAU). The neuronal cells were washed three times with PBS.

### Assessment of colocalization of CaM with RyR2 by immunocytochemistry

The CaM co-localized with RyR2 was detected by immunohistochemistry. The pixel-based colocalization of CaM and RyR2 was evaluated by the Pearson Correlation Coefficient and Manders’ Overlap Coefficient calculated using ImageJ/Fiji software with Coloc 2 plug-in.

### Preparation of brain homogenate

Whole brain homogenate prepared from 20-week-old mice was obtained according to Baghirova et al.^50^. Briefly, frozen mouse tissue was thawed and then minced into 2–4 mm pieces and washed with 1 mL ice-cold PBS. Then, 40–60 mg tissue was added into 500 μL ice-cold lysis buffer A. The tissue was disrupted in a tissue homogenizer and was centrifuged at 500×*g* for 10 min at 4 °C. The pellet was resuspended in lysis buffer A, followed by centrifugation at 4000×*g* for 10 min at 4 °C. The pellet was resuspended in lysis buffer B, followed by centrifugation at 6000×*g* for 10 min at 4 °C. The pellet was then used for Western blotting and CaM-SANPAH crosslinking experiments.

Lysis buffer A: NaCl 150 mM, HEPES (pH 7.4) 50 mM Digitonin 25 μg/mL, Hexylene glycol 1 M, Protease inhibitor cocktail 1% v:v.

Lysis buffer B: NaCl 150 mM, HEPES (pH 7.4) 50 mM, Igepal 1% v:v, Hexylene glycol 1 M, Protease inhibitor cocktail 1% v:v.

### Analysis of the binding characteristics of CaM to RyR2 with the CaM-SANPAH crosslinking method

Binding of CaM to RyR2 was evaluated using the photoreactive cross-linker, sulfosuccinimidyl-6-[4′-azido-2′-nitrophenylamino]hexanoate (Sulfo-SANPAH, Thermo Fisher Scientific, Waltham, USA), as described previously^11^. First, we made a CaM-SANPAH conjugate by mixing 50 μM recombinant CaM in conjugation buffer (150 mM KCl and 20 mM MOPS at pH 7.2) with 100 μM sulfo-SANPAH in the dark for 30 min. Conjugation was quenched by adding excess amount of lysine. CaM-SANPAH conjugate was purified using Amicon Ultra (MWCO 10 k). Mouse brain homogenates were diluted in binding buffer (150 mM KCl, 10 μM CaCl_2_, and 20 mM MES at pH 6.8) to 1 mg/mL and mixed with 100 nM CaM-SANPAH conjugate in the dark in a glass tube with or without CaMBPs. After a 10-min binding time, 30 s UV crosslinking was performed. Then, sample buffer was added to the crosslinked SR membrane followed by western blotting with anti-CaM (Merck, Millipore, Darmstadt, Germany). CaM-SANPAH crosslinked to RyR2 was detected as a 550 kDa band.

### Tissue fixation and preparation of paraffin sections

Brain hemispheres prepared from 20- and 40-week-old mice were fixed by immersion in 4% paraformaldehyde in phosphate buffer solution (Nacalai tesque, Kyoto, Japan). Fixed brains were cut every 1.5 mm and embedded in paraffin, and 4 µm thick sections were mounted onto glass slides. Sections were stained with hematoxylin and eosin (H&E) or were used for immunofluorescence.

### Immunohistofluorescence of Aβ and NeuN

As described by Wu et al.^41^, each brain of 20- and 40-week-old mice was fixed in 4% paraformaldehyde overnight at 4 °C prior to paraffin embedding. Coronal brain sections of 10 μm thickness were deparaffinized and hydrated through a series of graded alcohol steps and washed in PBS. Antigen retrieval was performed by heating in Antigen Unmasking Solution (Vector Labs, Burlingame, USA) in a decloaking chamber (Biocare Medical, Concord, USA) for 2 min. Sections were incubated in 5% hydrogen peroxide to block endogenous peroxidase activity. Sections were incubated overnight at 4 °C with the anti-β-amyloid 6E10 (1/600, Covance, Dedham, USA) and anti-NeuN EPR12763 (1/1500, Abcam, Cambridge, UK) in 1% BSA and 0.5% Triton X-100, followed by labeling with an Alexa 488-conjugated secondary antibody (1/300). The sections were washed three times with PBS.

### Neuronal cell density

NeuN immunostained, 3 μm thick brain slices were observed with a fluorescent digital microscope (BZ9000, Keyence, Japan, Plan 20 × Objective, Nikon, Japan), and all NeuN positive cells in the DG and CA areas of the entire hippocampus were counted and divided by the area of each region to calculate cell density.

### Western blot for NeuN

Hippocampal homogenates prepared from 20 and 40 week old mice were obtained, as described by Baghirova et al.^50^. Briefly, frozen mouse tissue was thawed and then minced into 2–4 mm slices and washed with 1 mL ice-cold PBS. Then, hippocampal tissue was obtained added into 500 μL ice-cold lysis buffer A supplemented with digitonin and protease inhibitor cocktail. The tissue was disrupted in a tissue homogenizer and was centrifuged at 500×*g* for 10 min at 4 °C. The pellet was resuspended in lysis buffer A followed by centrifugation at 4000×*g* for 10 min at 4 °C. The pellet was resuspended in lysis buffer B containing igepal followed by centrifugation at 6000×*g* for 10 min at 4 °C. The pellet was denatured in SDSPAGE sample buffer. SDS-PAGE, blotting, and antibody detections were performed with anti-NeuN EPR12763 (1/1500, Abcam, Cambridge, UK).

### Quantification of Aβ1–42 in mouse brain

Total proteins from whole brain of mice were extracted with lysis buffer. Then, Aβ1–42 was measured by an ELISA kit (WAKO-Fujifilm, Japan). In brief, protein solution diluted in PBS was placed into a 96-well micro plate coated with anti- Aβ1–16 antibody and incubated at 4 °C overnight. After washing, the sample was incubated for 1 h in 100 μl of horseradish peroxide-conjugated anti-human Aβ1-42 antibody. The sandwiched Aβ1–42 was visualized with a TMB solution.

### Statistics

Student *t* tests were used for statistical comparisons between two different conditions, whereas one-way or two-way ANOVA with a post-hoc Tukey’s or Dunnett test was used for statistical comparison of more than two groups. All data were expressed as means ± SE. A probability value of less than 0.05 was considered statistically significant.

### Ethics approval

All methods in this study conformed to the Guide for the Care and Use of Laboratory Animals published by the US National Institutes of Health (NIH Publication No. 85–23, revised 1996) and ARRIVE guidelines (http://www.nc3rs.org.uk/page.asp?id=1357). The care of animals and protocols used in this study were in accordance with guidelines from the Animal Ethics Committee of Yamaguchi University School of Medicine. This study was approved by the Animal Use Review Committee of Yamaguchi University.

## Supplementary Information

Supplementary Figures.
