# Supplementary material for: Enhancing calmodulin binding to ryanodine receptor is crucial to limit neuronal cell loss in Alzheimer disease
Source: Sci Rep. 2021 Mar 31;11:7289. doi: 10.1038/s41598-021-86822-x (PMC8012710; doi:10.1038/s41598-021-86822-x)
Supplement: Supplementary file 1 — Supplementary Figures. [file 41598_2021_86822_MOESM1_ESM.pdf]

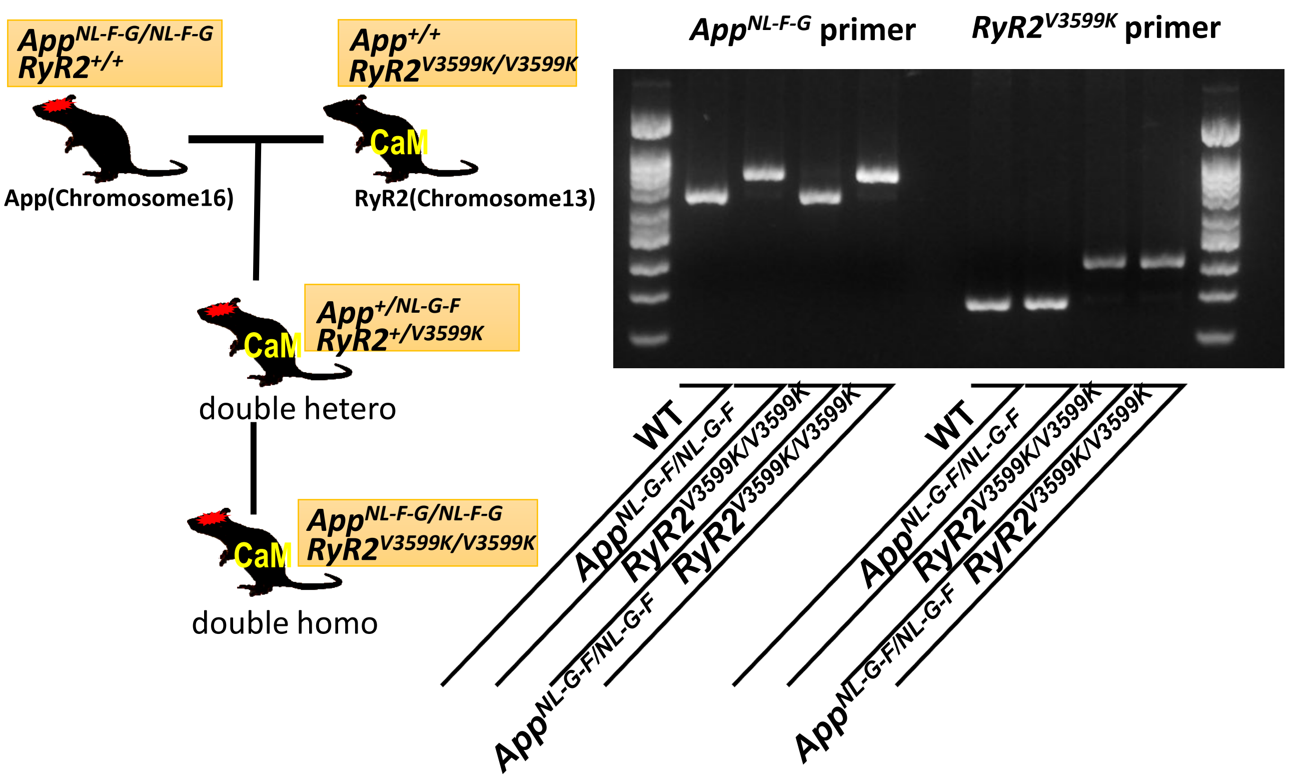


**Supplementary Fig. 1**

**Generation of $App^{NL\text{-}G\text{-}F}$ /RyR2 $^{V3599K}$ double homozygous mice**

A) $RyR2^{V3599K/V3599K}$ homozygous mice were crossed with $App^{NL\text{-}G\text{-}F/NL\text{-}F\text{-}G}$ homozygous mice. Generated $App^{+/NL\text{-}F\text{-}G}/RyR2^{+/V3599K}$ double heterozygous mice were crossed with each other to produce $App^{NL\text{-}G\text{-}F}/RyR2^{V3599K}$ double homozygous mice that were genotyped with polymerase chain reaction (PCR).

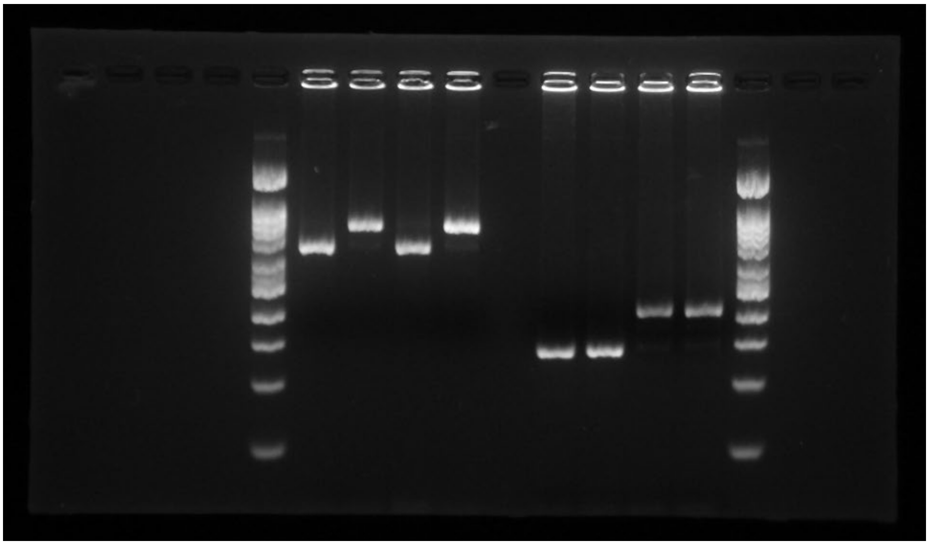

**Supplementary Fig. 1**

**Generation of $App^{NL\text{-}G\text{-}F}$ /RyR2 $^{V3599K}$ double homozygous mice**

B) Full-length gel shown in Supplementary figure 1A.

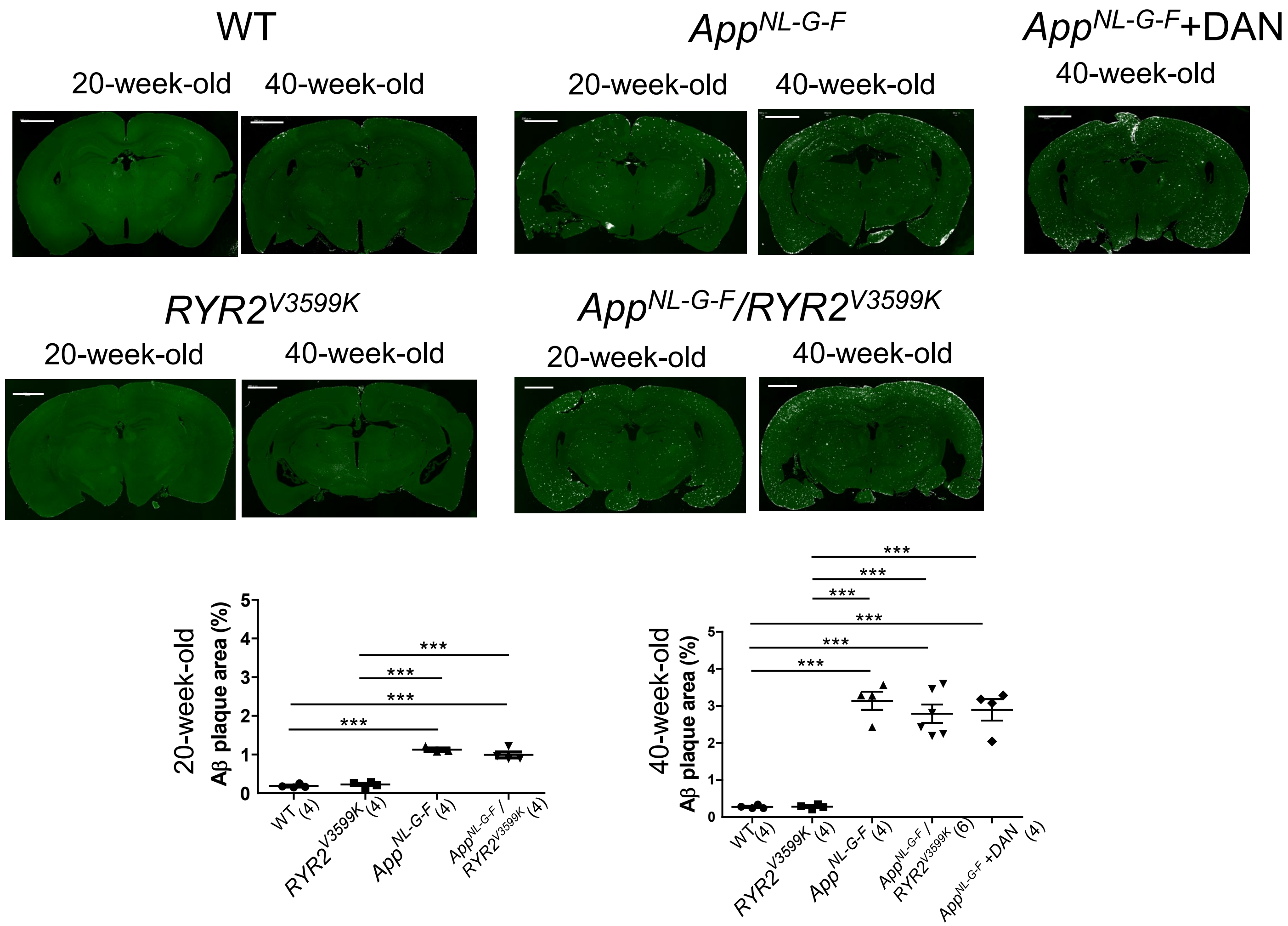


**Supplementary Fig. 2**

**Aβ in whole brain**

Amyloid beta (Aβ) in a cross section of the brain and the summarized data. Aβ was evaluated by immunocytochemistry using anti-Aβ. Aβ was expressed as an area (%) normalized by the whole brain area. Scale bars: 1000 μm. N=4-6 mice. Parentheses indicate the number of brain. ***$p < 0.001$ (one-way ANOVA with post-hoc Tukey's multiple comparison test).

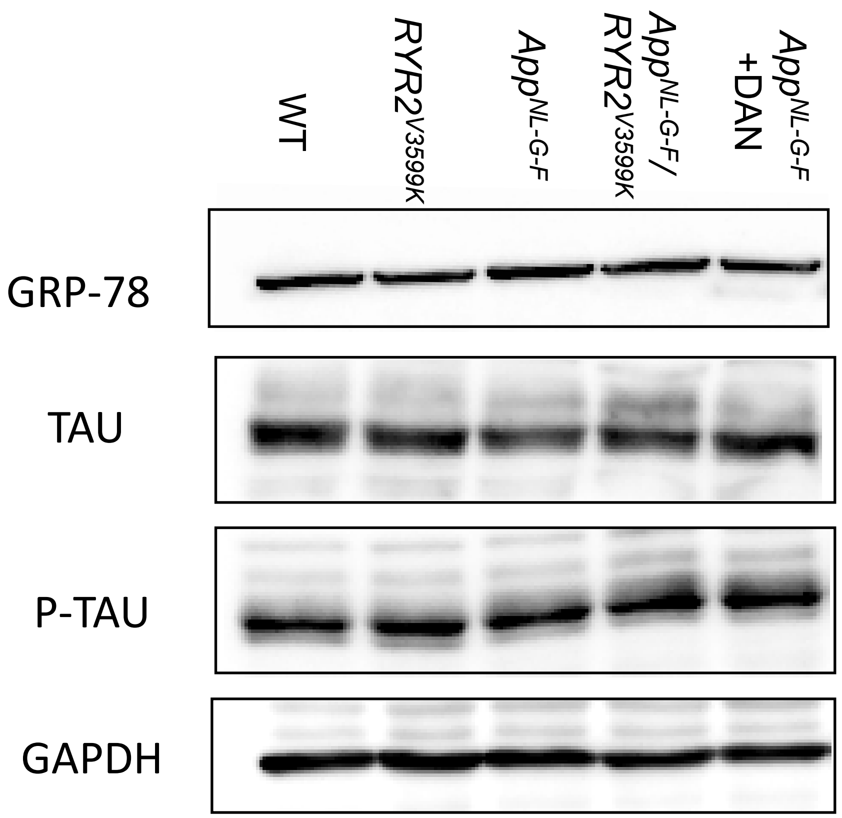


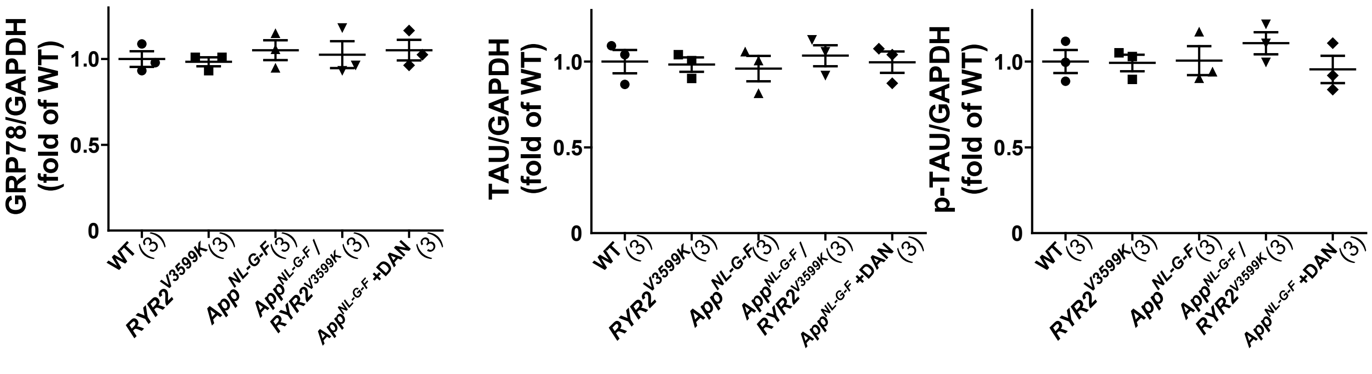


Supplementary Fig. 3

**A) Expression of ER stress marker and p-TAU in brain homogenate of 40-week-old mice**

(Top) Western blots of GRP-78, TAU and p-TAU. (Bottom) Summarized data of western blotting. Data are presented as mean ± SE of 3 mouse.

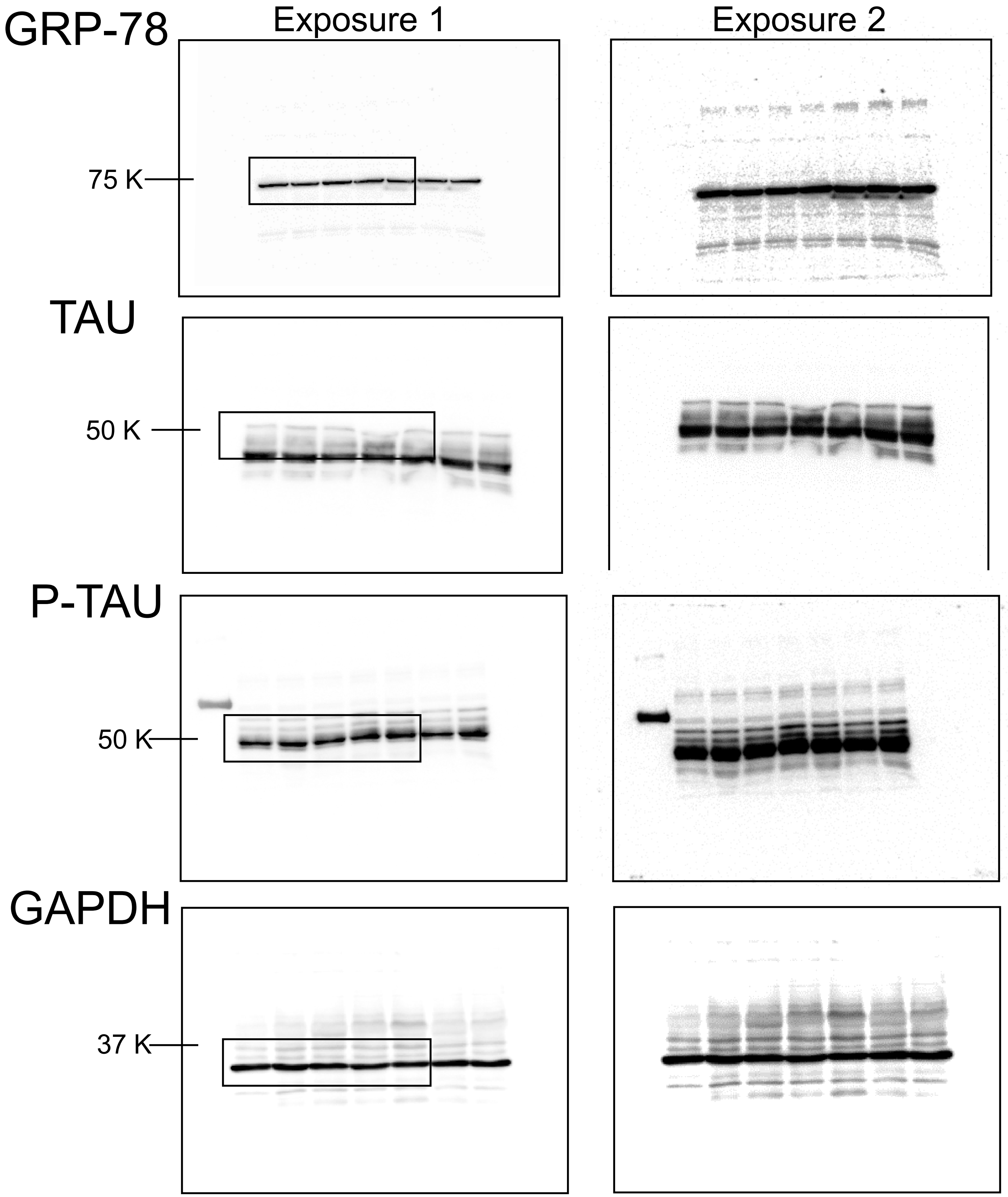


Supplementary Fig. 3

**B) Full-length uncropped membranes of Western blots**

We presented two types of exposure and the bands on the figure were enclosed.

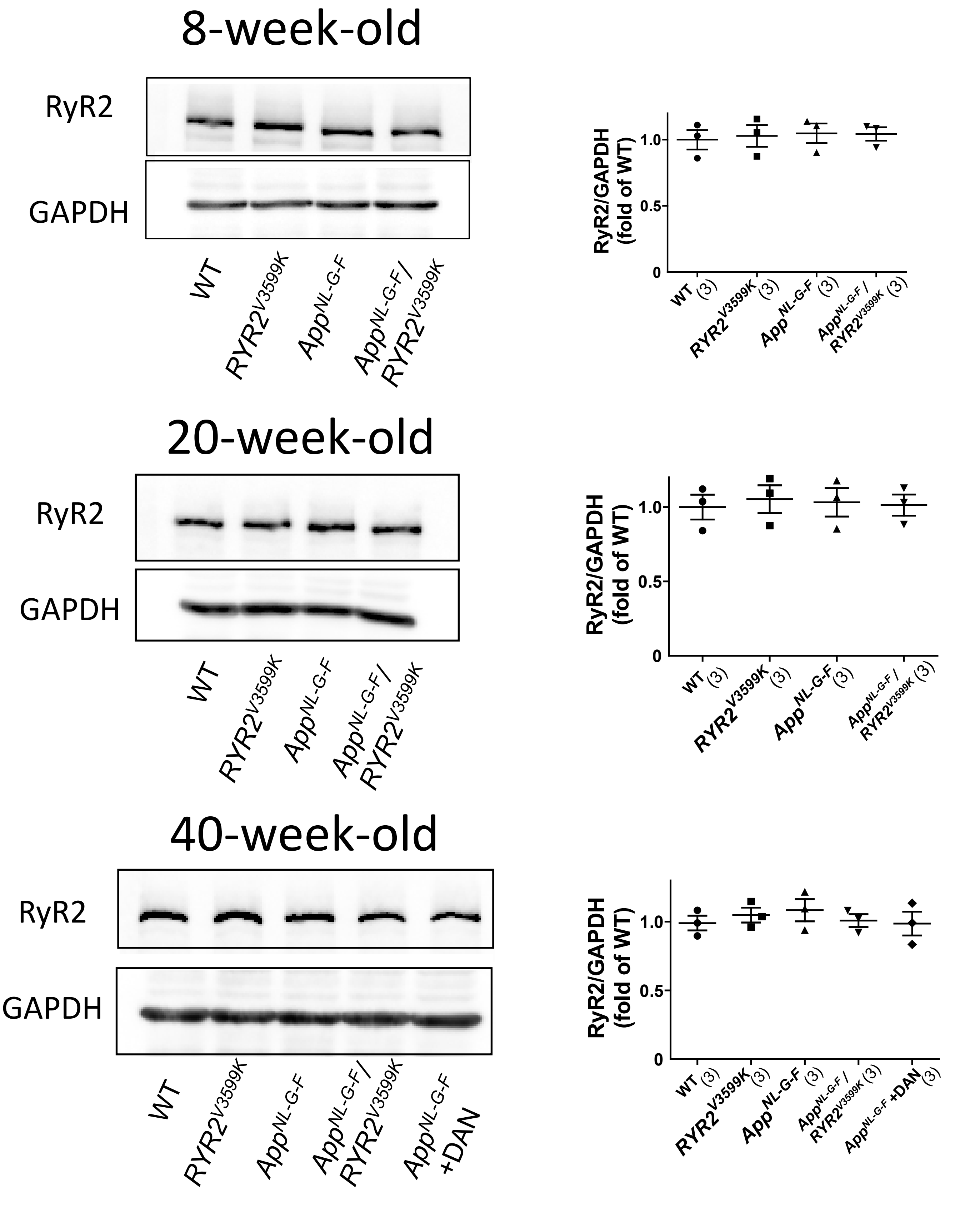


**Supplementary Fig. 4**

**A) RyR2 expression levels in brain homogenate of 8-, 20- and 40-week-old mice**

(left) Western blots of RyR2 and GAPDH, glyceraldehyde-3-phosphate dehydrogenase. (right) Summarized data of western blotting. Data are presented as mean ± SE of 3 mouse.

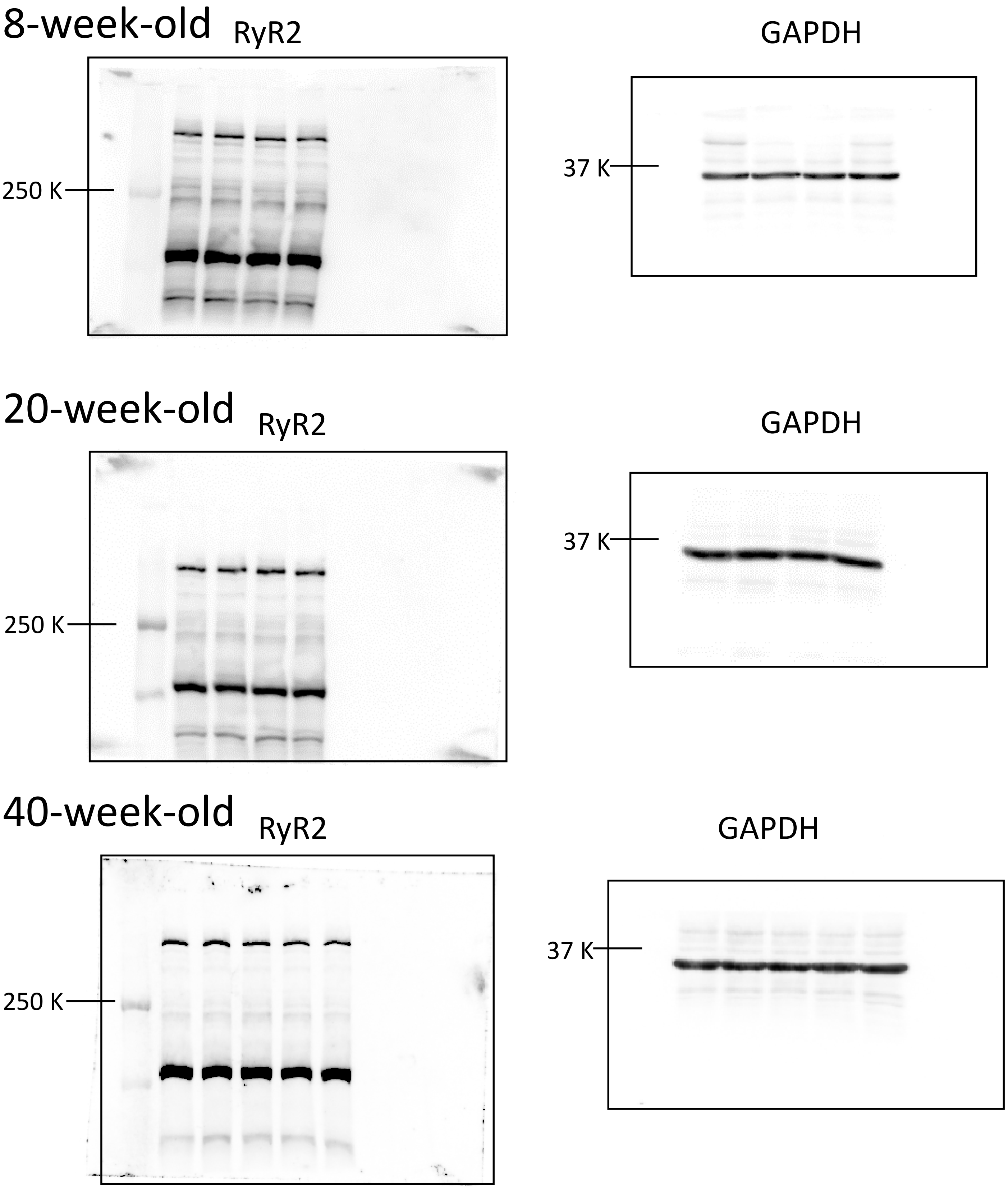


Supplementary Fig. 4

B) Full-length uncropped membranes of Western blots

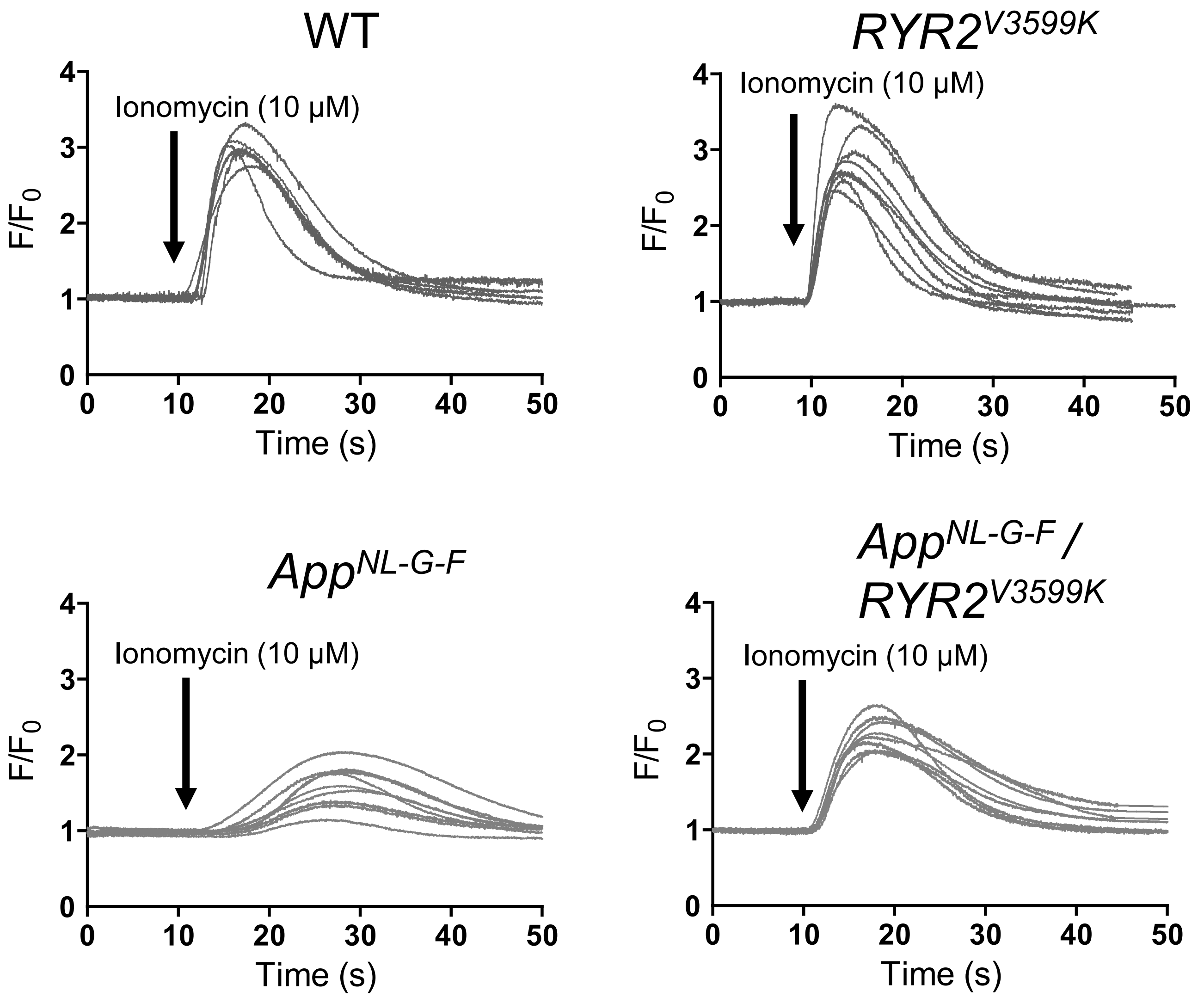


**Supplementary Fig. 5**

**Time course of intracellular $Ca^{2+}$ in response to ionomycin, in WT, RyR2V3599K, AppNL-G-F, and AppNL-G-F/RYR2V3599K neuronal cells.**

Representative images of Cal520 fluorescence changes evoked by ionomycin (10 μM) from 8 - 9 cells of 3 mice.

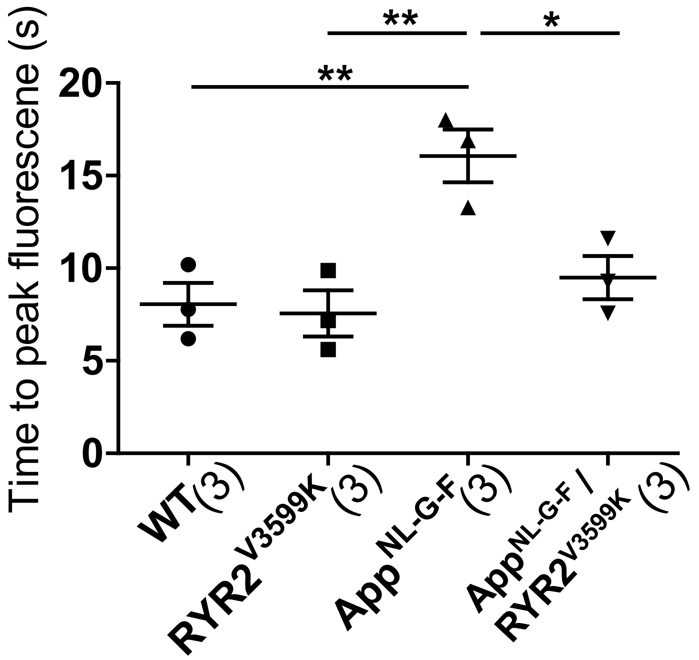


**Supplementary Fig. 5**

**Time course of intracellular $Ca^{2+}$ in response to ionomycin, in WT, RyR2V3599K, AppNL-G-F, and AppNL-G-F/RYR2V3599K neuronal cells.**

Summarized data of time to peak fluorescence intensity. Values for individual mice are plotted together with mean ± SEM. N= 21-31 cells from 3 mice. Parentheses indicate the number of mice. $*p < 0.05$, $**P < 0.01$ (one-way ANOVA with post-hoc Tukey's multiple comparison test)

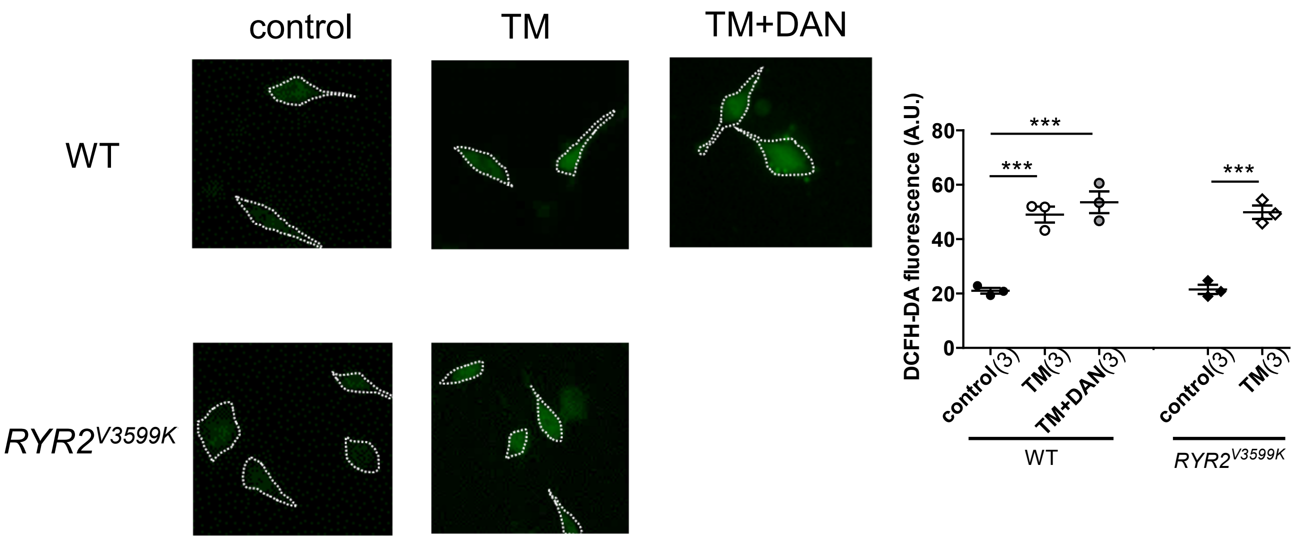


**Supplementary Fig. 6**

**DCFH-DA fluorescence change after addition of TM in neuronal cells.**

DAN: dantrolene. Values for individual mice are plotted together with mean ± SEM. N= 57-72 cells from 3 mice. Parentheses indicate the number of mice. ***p < 0.001 (one-way ANOVA with post-hoc Tukey's multiple comparison test).

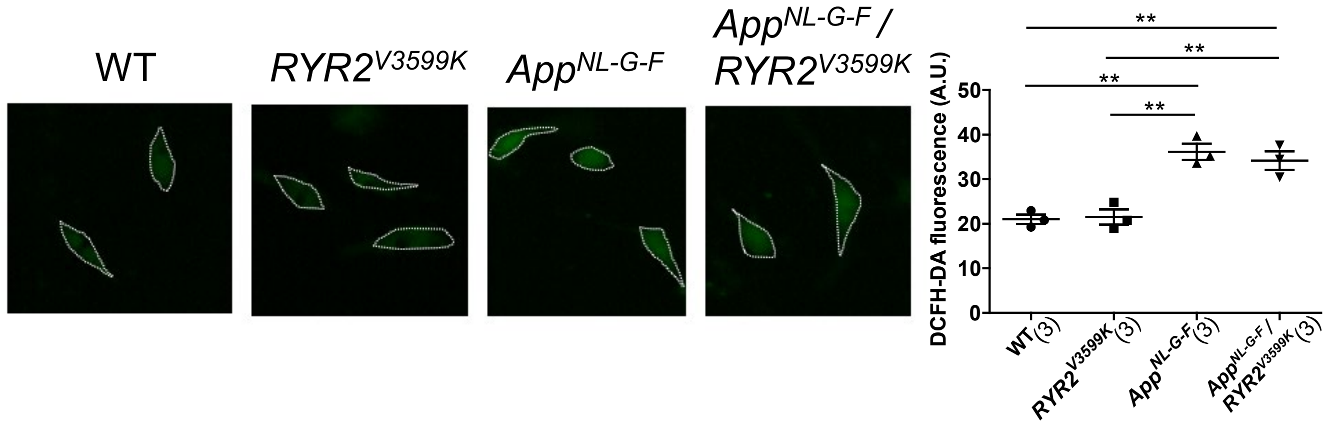


**Supplementary Fig. 7**

**DCFH-DA fluorescence in WT, *App*$^{NL\text{-}G\text{-}F}$, *RYR2*$^{V3599K}$, and *App*$^{NL\text{-}G\text{-}F}$/*RYR2*$^{V3599K}$ neuronal cells.**

Values for individual mice are plotted together with mean ± SEM. N=69 - 93 cells from 3 mice. Parentheses indicate the number of mice. **p < 0.01(one-way ANOVA with post-hoc Tukey's multiple comparison test).

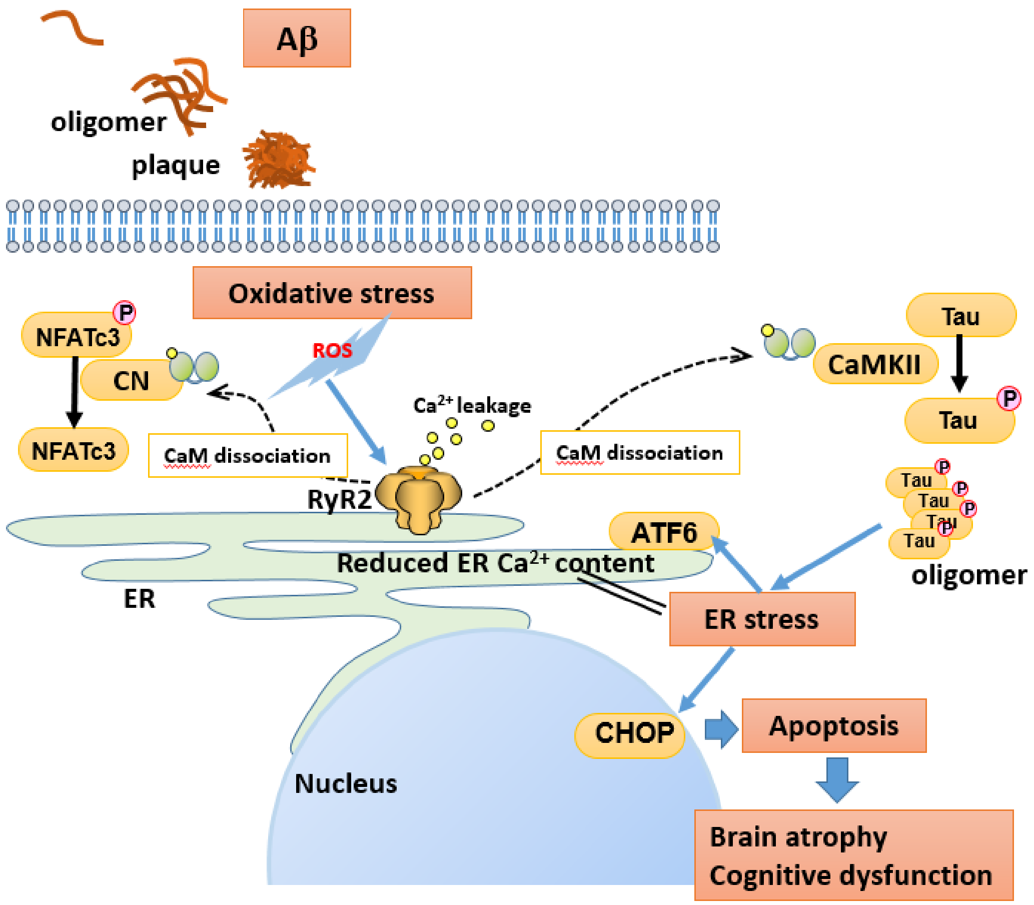


Supplementary Fig. 8

**Novel therapeutic strategy to limit neuronal cell loss in Alzheimer's disease.**

**A) Postulated mechanism of $Ca^{2+}$ dysregulation in Alzheimer's disease.** Mutation in amyloid precursor protein produces soluble amyloid-β oligomers (Aβ) which may increase oxidative stress. Oxidative stress induces domain unzipping between N-terminal (aa 1-600) and central domains (aa 2000-2500) in RyR2, which allosterically displaces CaM from RyR2, thereby causing ER $Ca^{2+}$ leakage. Owing to $Ca^{2+}$ leakage, ER $Ca^{2+}$ content decreases, which in turn induces ER stress, TAU phosphorylation, and apoptosis, thereby resulting in neuronal cell loss and cognitive dysfunction. Both CaMKII and calcineurin, by binding $Ca^{2+}$-CaM originated from RyR2, accelerate Tau phosphorylation. ROS: reactive oxygen species, CaM: calmodulin, RyR2; ryanodine receptor 2, CaMKII: $Ca^{2+}$ /calmodulin-dependent protein kinase II, CaN: calcineurin, ER: endoplasmic reticulum, GRP78: Glucose-Regulated Protein, 78kD, ATF6: Activating Transcription Factor 6, CHOP: C/EBP-homologous protein.

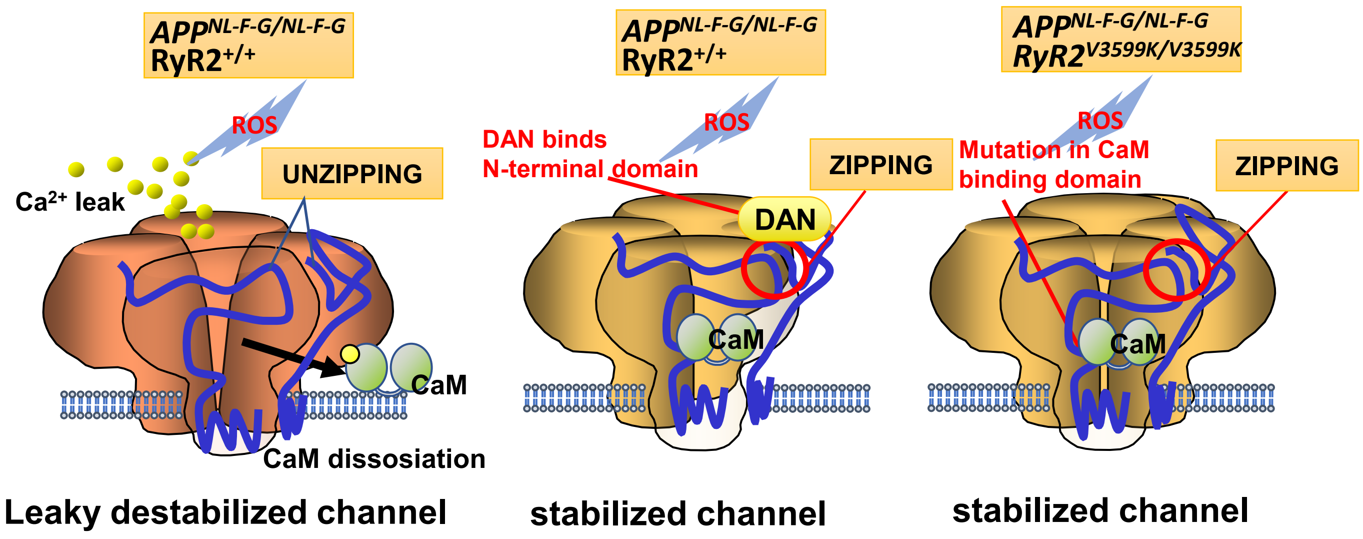


Supplementary Fig. 8

**Novel therapeutic strategy to limit neuronal cell loss in Alzheimer's disease.**

**B) Stabilizing RyR2 by enhancing CaM binding to RyR2, thereby ameliorating AD.** Dantrolene specifically binds to the N-terminal domain (aa 601-620) of RyR2 and shifts the mode from the unzipped to zipped state in the N-terminal (aa 1-600) and central (aa 2000-2500) domain-domain interactions, thereby preventing CaM dissociation and subsequent $Ca^{2+}$ leakage. V3599K mutation in CaM binding domain (aa 3584-3603) markedly enhances CaM binding affinity to RyR2, thereby inhibiting $Ca^{2+}$ leakage, ER stress and apoptosis.

WT
(control)

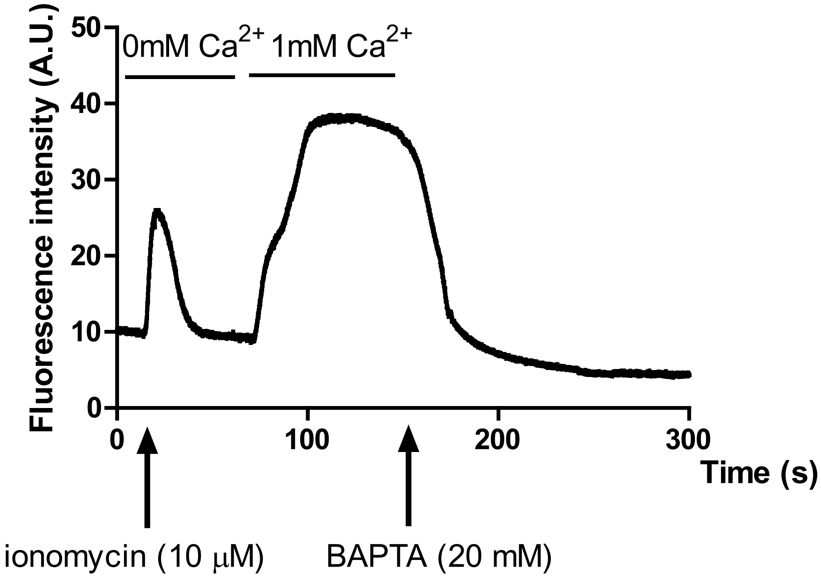


RYR2 V3599K
(control)

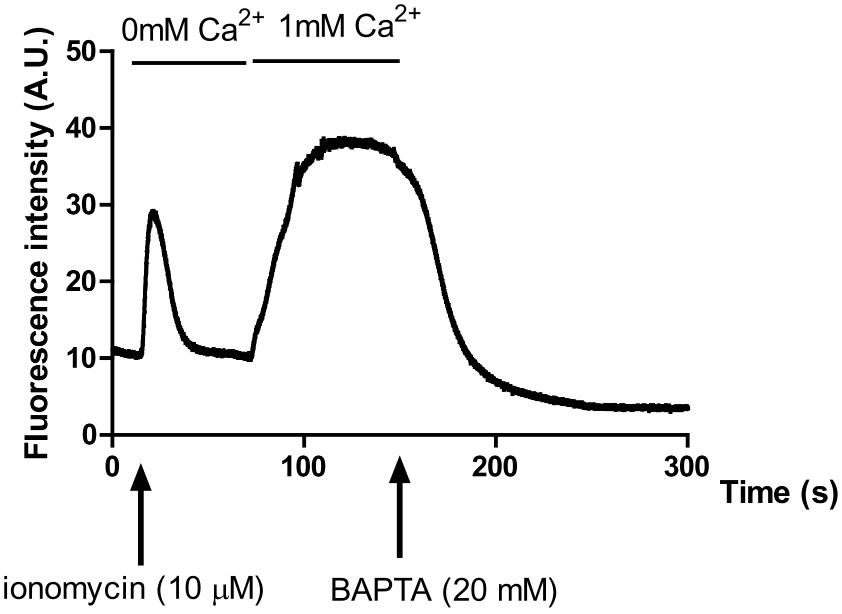


**Supplementary Fig. 9**

**The whole process of intracellular $Ca^{2+}$ monitoring**

Cal520 with a Kd of 320 nM was used. Cal520 AM was loaded into isolated neurons in calcium-free Tyrode's solution (140 mM NaCl, 6 mM KCl, 1 mM $MgCl_2$, 10 mM glucose, and 5 mM HEPES-Tris, pH 7.4). After confirming the reaction of ionomycin, 1mM $Ca^{2+}$ was added to measure Fmax, and subsequently 20mM BAPTA was added to measure Fmin. The absolute $Ca^{2+}$ concentrations was calculated from the following formula. $Ca^{2+}$ = Kd (F – Fmin) / (Fmax – F); Kd = 320 nM.

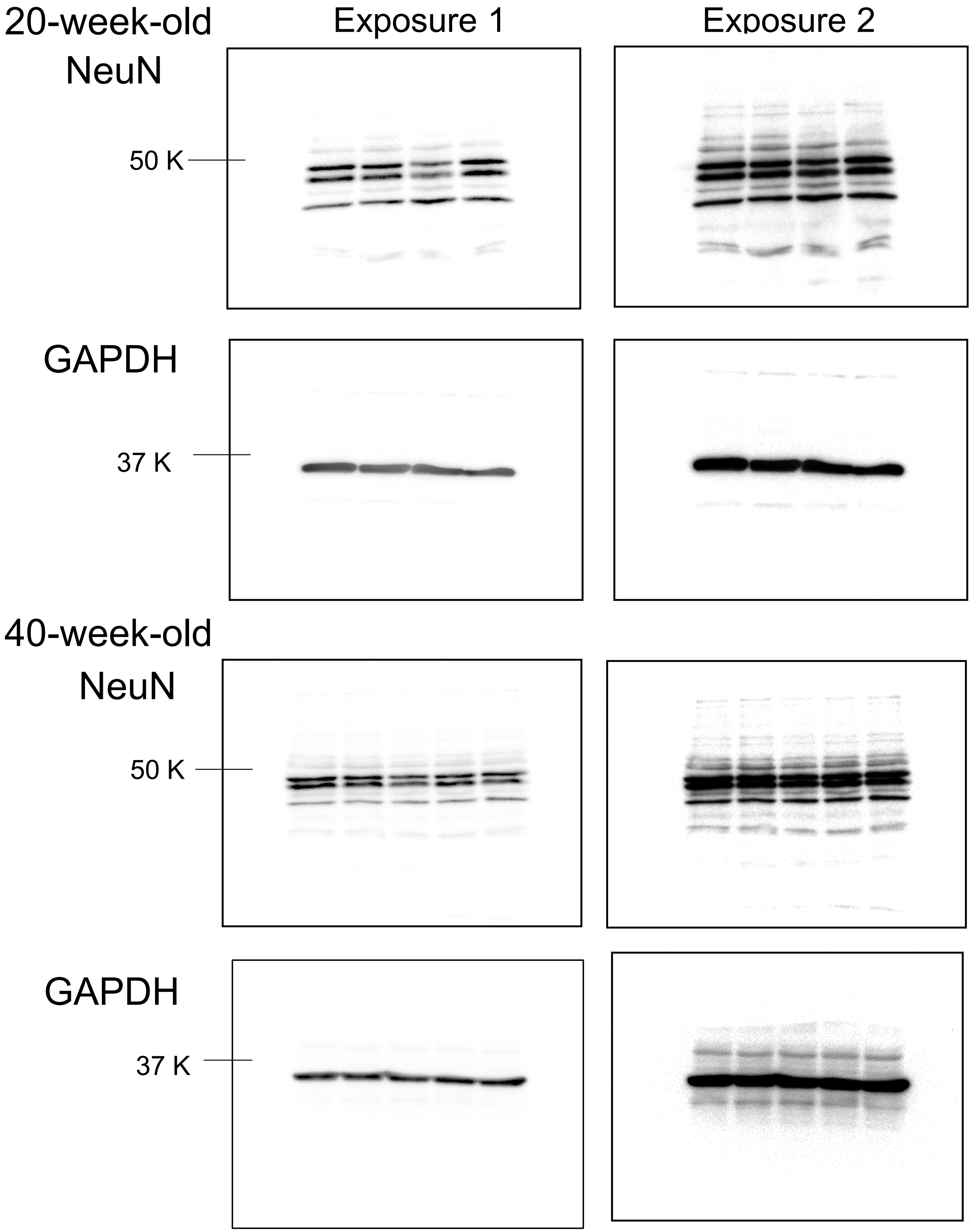


**Supplementary Fig. 10**

**Full-length uncropped membranes of Western blots for NeuN and GAPDH shown in Figure 1E**We presented two types of exposure.

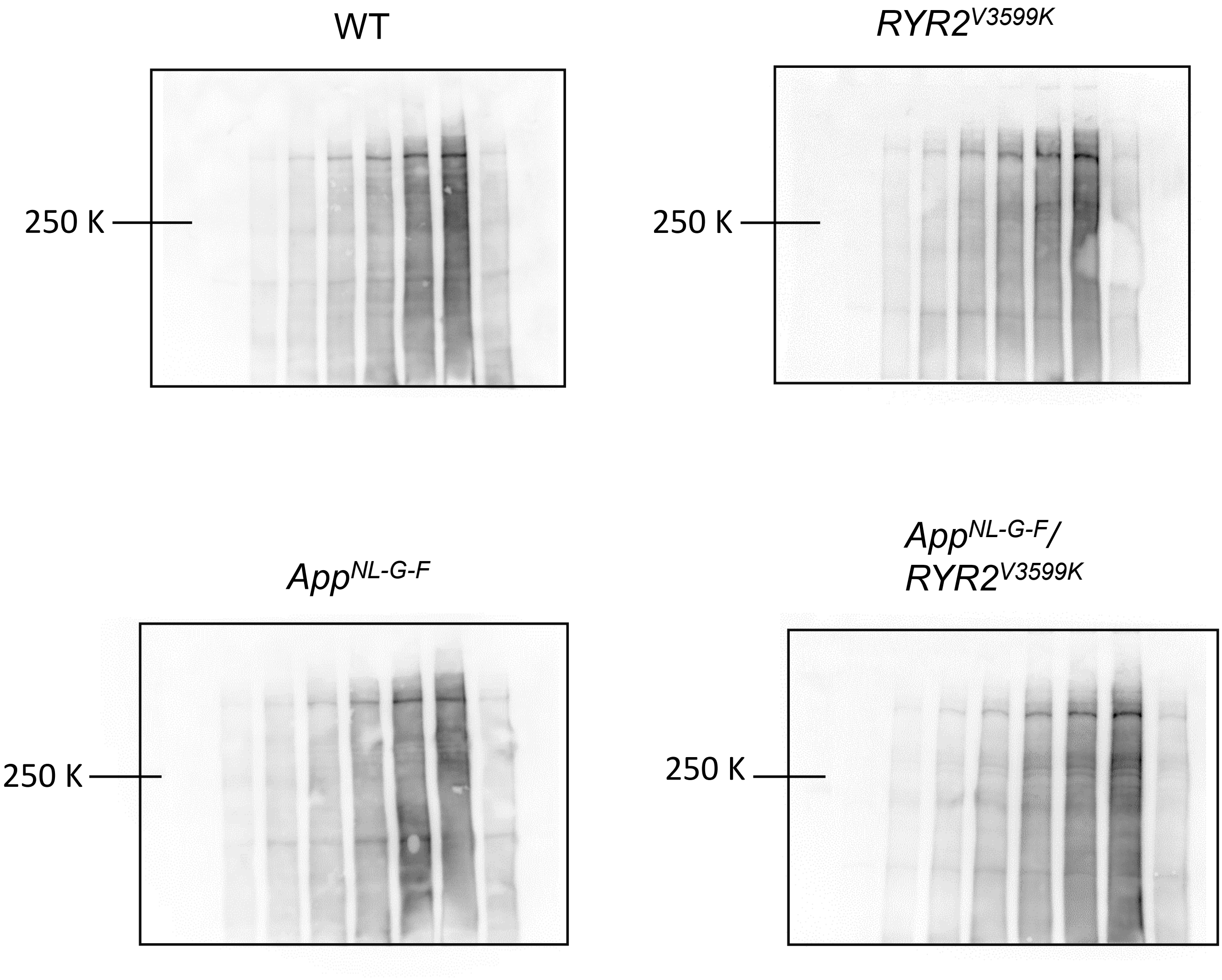


**Supplementary Fig. 11**

**Full-length uncropped membranes of Western blots for RyR2-bound CaM-SANPAH shown in Figure 3B**

The bands were detected by anti-CaM antibody (Merck, Millipore, Darmstadt, Germany) and RyR2-bound CaM-SANPAH was detected as a 550 kDa band.

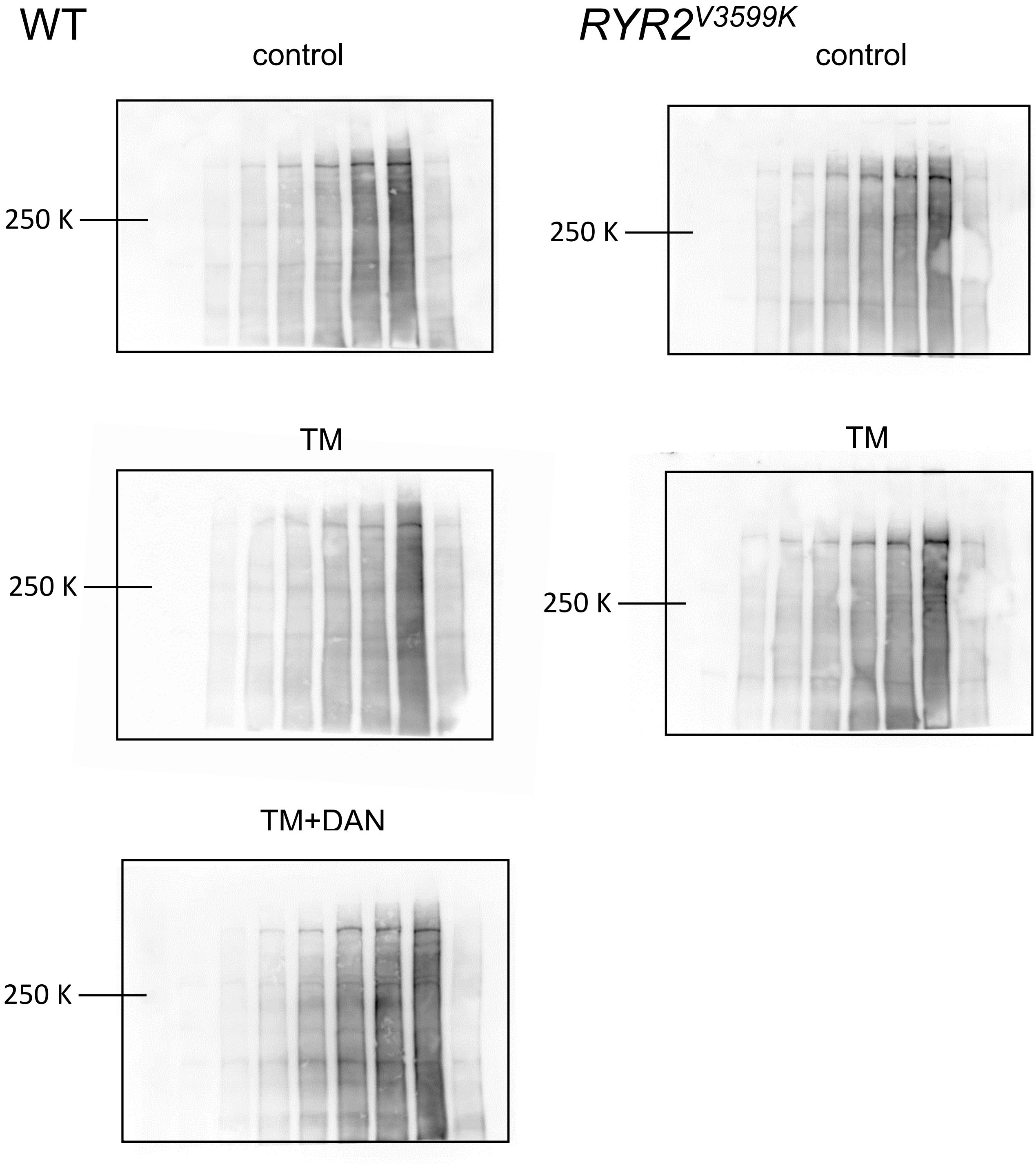


**Supplementary Fig. 12**

**Full-length uncropped membranes of Western blots for RyR2-bound CaM-SANPAH shown in Figure 4D**

The bands were detected by anti-CaM antibody (Merck, Millipore, Darmstadt, Germany) and RyR2-bound CaM-SANPAH was detected as a 550 kDa band.
